# Mating‐Type Loci Modulate Pathogenicity and Non‐Sexual Development Through Autocrine Pheromone Signalling in the Asexual Fungus *Fusarium oxysporum*

**DOI:** 10.1111/mpp.70248

**Published:** 2026-03-23

**Authors:** Stefania Vitale, Antonia Barberio, Riccardo Cantelli, Marta Ranesi, Filippo De Curtis, Antonio Di Pietro, David Turrà

**Affiliations:** ^1^ Department of Agricultural Sciences University of Naples Federico II Portici Italy; ^2^ Departamento de Genética, Campus de Excelencia Internacional Agroalimentario ceiA3 Universidad de Córdoba Córdoba Spain; ^3^ Bioelectronics Task Force University of Naples Federico II Naples Italy; ^4^ Department of Agricultural, Environmental and Food Sciences University of Molise Campobasso Italy; ^5^ Center for Studies on Bioinspired Agro‐Environmental Technology University of Naples Federico II Portici Italy; ^6^ Computational and Quantitative Biology Task Force University of Naples Federico II Naples Italy

**Keywords:** autocrine pheromone signalling, evolutionary co‐option, fungal pathogenicity, *Fusarium oxysporum*, mating‐type loci, quorum sensing

## Abstract

Mating‐type (*MAT*) loci are traditionally considered vestigial remnants in asexual fungi, yet their widespread retention suggests additional, yet unrecognised functions. Here we show that in the asexual plant pathogen *Fusarium oxysporum* f.sp. *lycopersici* the two *MAT* loci function as master regulators of developmental processes through autocrine pheromone signalling. *MAT1‐1* and *MAT1‐2* exhibit opposing regulatory roles in density‐dependent conidial germination, creating a bistable switch for population‐level behavioural coordination. *MAT1‐1* promotes vegetative hyphal fusion and multicellular aggregation, whereas *MAT1‐2* inhibits these processes. These opposing effects are mediated in part by enhanced expression of the protease Bar1 in *MAT1‐2* isolates, which specifically cleaves α‐pheromone thereby modulating signalling responses. Unexpectedly, *MAT1‐1* enhances virulence of *F. oxysporum* on tomato plants in a background‐dependent manner, whereas *MAT1‐2* exhibits only a slight influence on pathogenicity. Together, our findings establish that *MAT* loci have undergone evolutionary repurposing to control essential developmental processes through autocrine communication networks, revealing novel targets for sustainable disease management approaches.

## Graphical abstract

*MAT* loci function as bistable switches in the asexual plant‐pathogenic fungus *Fusarium oxysporum*, controlling population behaviour through autocrine pheromone signalling. *MAT1‐1* promotes hyphal fusion and virulence while repressing germination. *MAT1‐2* has opposite effects, upregulating Bar1 protease that cleaves α‐pheromone. This enables density‐dependent coordination of development and infection.

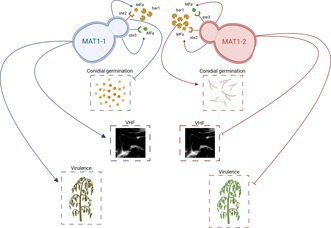

## 1 Introduction

Sexual mating‐type (*MAT*) loci represent sophisticated regulatory systems in fungal biology. These loci orchestrate complex transcriptional networks that control pheromone‐receptor signalling pathways during sexual reproduction across ascomycetous fungi (Heitman et al. 2013; Fraser and Heitman 2005). *MAT* loci exist as idiomorphs—structurally unrelated sequences occupying homologous positions. They encode transcription factors with highly conserved DNA‐binding domains that function as master switches controlling sexual identity and reproductive compatibility (Debuchy and Turgeon 2006; Turgeon and Yoder 2000). In ascomycetes, the *MAT1‐1* idiomorph harbours genes encoding proteins with α‐box (*MAT1‐1‐1*), proline‐proline‐phenylalanine (PPF) (*MAT1‐1‐2*) and high‐mobility group (HMG) domains (*MAT1‐1‐3*), whereas the *MAT1‐2* idiomorph contains genes encoding HMG DNA‐binding proteins (*MAT1‐2‐1*) and accessory factors (*MAT1‐2‐9*). This complementary regulatory architecture prevents self‐fertilisation while enabling outcrossing through sophisticated mate recognition systems (Bennett and Johnson 2005; Martin et al. 2011; Ni et al. 2011; Montoya‐Martínez et al. 2019). In heterothallic species, sexual reproduction requires outcrossing between individuals carrying different *MAT* idiomorphs (*MAT1‐1* or *MAT1‐2*), ensuring genetic recombination. In contrast, homothallic species possess both *MAT* idiomorphs within a single genome, enabling self‐fertility and sexual cycle completion within an individual isolate (Yun et al. 2000).

The evolutionary maintenance of a complex sexual machinery across diverse fungal lineages, some of which lack a known sexual cycle, suggests a fundamental role extending beyond reproductive control. Many economically critical plant pathogens reproduce exclusively through asexual mechanisms while retaining fully functional *MAT* loci. These loci maintain intact gene structures and active transcriptional machinery (Hartmann et al. 2021; Dyer and Kück 2017; Wallen and Perlin 2018). *Fusarium oxysporum* exemplifies this evolutionary contradiction. It represents one of the most devastating fungal pathogen complexes worldwide that lacks a described sexual cycle. However, *F. oxysporum* possesses *MAT* loci with genomic organisation and transcriptional architecture virtually identical to heterothallic sexually reproducing relatives (Yun et al. 2000; Martin et al. 2011).

Recent studies demonstrated that fungal sex pheromones and receptors function in processes independent of sexual reproduction (Turrà et al. 2015; Vitale et al. 2019). Initial investigations revealed that *F. oxysporum* utilises pheromone receptors for chemotropic sensing of host plant signals, representing the first documented repurposing of sexual communication molecules for pathogen–host interactions (Turrà et al. 2015). Subsequent studies provided evidence for autocrine pheromone signalling (APS) in *F. oxysporum*, where individual cells of the same mating type simultaneously produce and sense a‐ and α‐factor pheromones via the cognate Ste3 and Ste2 receptors, thereby creating a self‐signalling loop that enables population‐level coordination of developmental decisions (Vitale et al. 2019).

APS functions via two parallel pheromone‐receptor pathways with opposing effects that enable precise cell density sensing (Vitale et al. 2019). Binding of α‐pheromone to the Ste2 receptor activates the Mpk1 MAPK cascade, which represses germination at high cell density. In contrast, a‐pheromone signalling through the Ste3 receptor alleviates this germination inhibition by competing for the same MAPK cascade. Such simultaneous co‐expression of all four APS components enables autocrine signalling within a single mating type (Vitale et al. 2019) but differs dramatically from canonical pheromone systems where mating‐type identity limits expression to one given peptide pheromone and the opposite pheromone receptor (Debuchy and Turgeon 2006; Turgeon and Yoder 2000).

Vegetative hyphal fusion (VHF) represents another fundamental developmental process bridging individual behaviour with multicellular organisation through specialised conidial anastomosis tubes (CATs), facilitating cytoplasmic continuity essential for nutrient sharing and colony‐wide communication (Read et al. 2009; Glass et al. 2004). In plant‐pathogenic fungi, VHF serves specialised functions in infection establishment and tissue colonisation, enhancing surface adhesion and facilitating coordinated invasive growth through formation of interconnected hyphal networks that optimise nutrient exploitation during host colonisation (Prados‐Rosales and Di Pietro 2008). Furthermore, hyphal aggregate formation represents an additional sophisticated multicellular coordination process involving the assembly of complex three‐dimensional biofilm‐like structures that are embedded within a self‐produced extracellular polymeric matrix. This creates a protective microenvironment that enhances pathogen survival under adverse conditions (Beauvais et al. 2007; Segorbe et al. 2017; Shopova et al. 2013). Such multicellular assemblages enable population‐level communication through quorum sensing and facilitate coordinated behaviour across fungal communities, supporting complex processes such as pathogenicity (Albuquerque and Casadevall 2012).

Contemporary research has recognised the central importance of transcriptional regulation in coordinating virulence factor expression and environmental adaptation, with over 700 predicted transcription factors in *F. oxysporum* representing enormous regulatory potential (Zuriegat et al. 2021). During infection, *F. oxysporum* must coordinate expression of diverse virulence factors including cell wall‐degrading enzymes, effector proteins and secondary metabolites, while responding to host defences and environmental insults (Zuriegat et al. 2021; Michielse and Rep 2009; Redkar et al. 2022; Gámez‐Arjona et al. 2022). The integration of population density with virulence functions enables pathogens to coordinate infection strategies based on local cell concentrations, optimising the timing of virulence factor deployment (Albuquerque and Casadevall 2012; Hornby et al. 2001).

Given the established role of *MAT* loci as master regulators controlling pheromone‐receptor networks essential for sexual reproduction, combined with mounting evidence of extensive evolutionary repurposing in fungal pathogens, we hypothesised that mating‐type regions serve as master regulatory hubs coordinating multiple aspects of fungal biology extending far beyond sexual reproduction (Fraser and Heitman 2004; Heitman et al. 2007; Turrà et al. 2015; Vitale et al. 2019). Here we used targeted deletion of *MAT* loci as well as engineered strains carrying both *MAT* idiomorphs to explore their role in cell density‐dependent germination, hyphal fusion, multicellular aggregation and pathogenicity of *F. oxysporum*. Our findings reveal a previously unknown role of *MAT* loci in this apparently asexual organism while providing mechanistic insights into the regulatory network coordinating development, environmental sensing and virulence in one of agriculture's most devastating fungal pathogens.

## 2 Results

### 2.1 *MAT* Locus Organisation and Generation of *MAT* Locus Mutants

**FIGURE 1:**
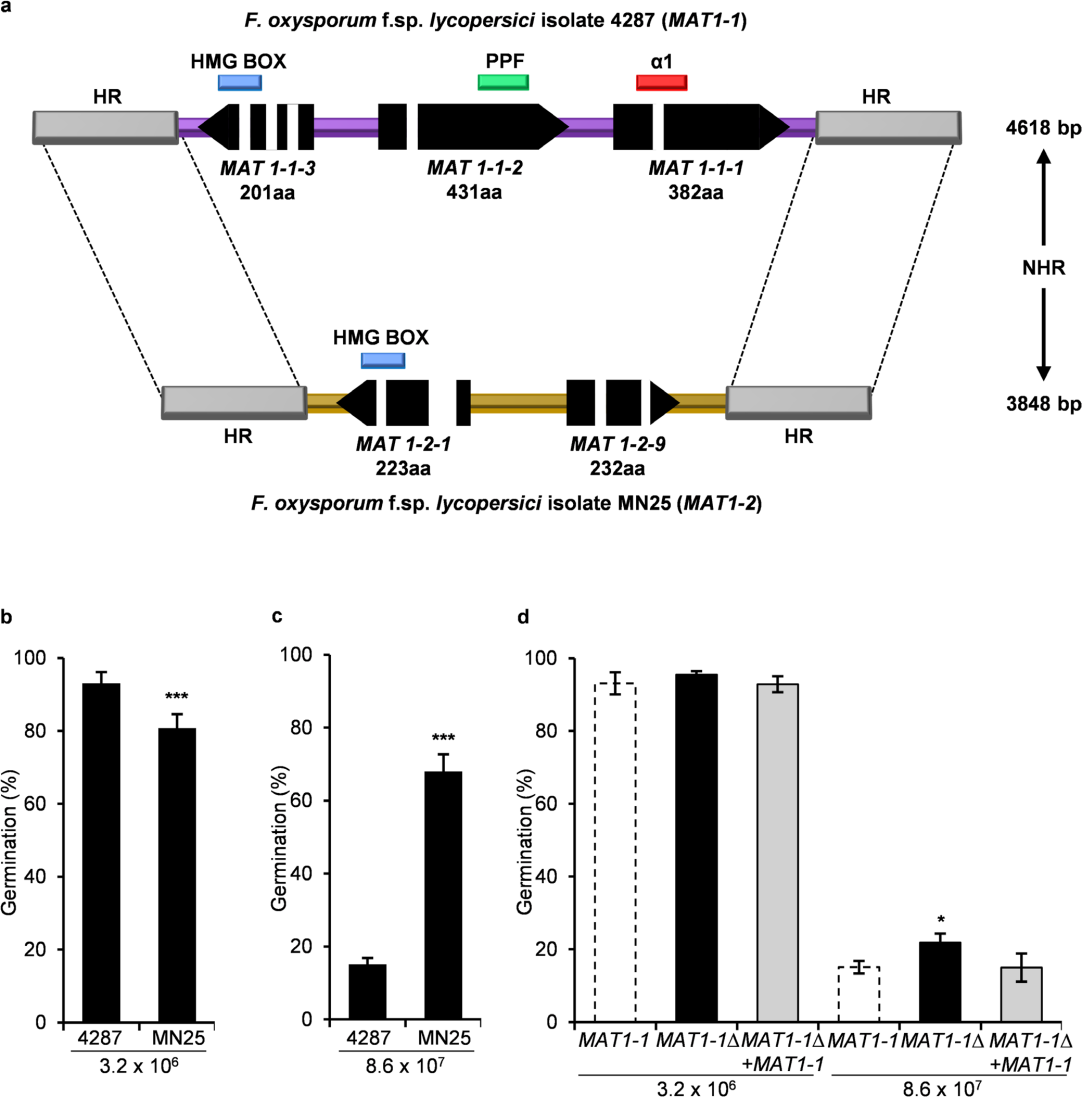
*MAT* loci function as opposing regulators of density‐dependent conidial germination. (a) Schematic representation of *MAT1‐1* and *MAT1‐2* locus organisation in *Fusarium oxysporum* f. sp. *lycopersici* isolates 4287 and MN25. Arrows indicate transcription direction, coloured boxes represent protein‐coding domains (α‐box, PPF, HMG), and HR indicates homologous regions (> 99% identity) flanking the idiomorphic sequences. (b) Germination rates at low concentration of inoculum (LCI; 3.2 × 10^6^ conidia/mL) showing normal spore viability across all strains with a decreased germination in the *MAT1‐2* isolate MN25 (***p < 0.001 vs. *MAT1‐1* isolate 4287). (c) Germination responses at high concentration of inoculum (HCI; 8.6 × 10^7^ conidia/mL) revealing *MAT* locus‐dependent regulation (***p < 0.001 vs. *MAT1‐1* isolate 4287). *MAT1‐1* isolate 4287 exhibits strong density‐dependent inhibition while *MAT1‐2* isolate MN25 maintains robust germination capacity. (d) Genetic complementation analysis confirming *MAT1‐1* function. *MAT1‐1* deletion (*MAT1‐1*Δ) significantly derepresses germination at high density (*p < 0.05 vs. *MAT1‐1* isolate), whereas complementation (*MAT1‐1*Δ + *MAT1‐1*) restores wild‐type inhibition. Data represent means ± standard deviation from three independent experiments (*n* = 300 conidia per replicate). *p* values were calculated using Yates' corrected chi‐squared test (two‐sided).

Sequence analysis of the two *F. oxysporum* f. sp. *lycopersici* isolates 4287 and MN25 confirmed that they carry the *MAT1‐1* and *MAT1‐2* idiomorphs, respectively, with a conserved *MAT* locus organisation typical of heterothallic *Fusarium* species (Figure 1a) (Montoya‐Martínez et al. 2019). The *MAT1‐1* locus in isolate 4287 spans 4.6 kb and contains three predicted genes: *MAT1‐1‐1* (*FOXG_03616*), *MAT1‐1‐2* (*FOXG_03615*) and *MAT1‐1‐3* (*FOXG_17746*). The *MAT1‐2* locus in isolate MN25 encompasses 3.8 kb and includes two predicted genes: *MAT1‐2‐1* (*FOWG_12730*) and *MAT1‐2‐9* (*FOWG_12731*).

The three genes in the *MAT1‐1* idiomorph of isolate 4287 encode proteins with specific, highly conserved domains: a central α‐box domain in *MAT1‐1‐1*, an invariant PPF domain in *MAT1‐1‐2* and an HMG domain in *MAT1‐1‐3*. In contrast, the *MAT1‐2* idiomorph of MN25 contains *MAT1‐2‐1* encoding a protein with an HMG domain and *MAT1‐2‐9* lacking conserved domains (Figure 1a). This distinct domain architecture, α‐box, PPF and HMG for *MAT1‐1* versus HMG alone for *MAT1‐2*, is a hallmark of *MAT* idiomorphs in the *Sordariomycetes* (Ni et al. 2011), to which *Fusarium* belongs. The presence of these canonical domains (Figure 1a) and the syntenic arrangement of the genes flanking the two *MAT* loci (data not shown) are consistent with the genomic organisation found in sexually reproducing fungi, suggesting a potential for a sexual cycle which has not been observed so far in *F. oxysporum*.

Genome sequence analysis in isolates 4287 and MN25 revealed > 99% sequence identity in the regions flanking the *MAT1‐1* and *MAT1‐2* loci, suggesting the presence of homologous recombination sites suitable for targeted replacement (Figure 1a). Using the split‐marker strategy, we generated *MAT1‐1* deletion mutants in the 4287 isolate (Figure S1a–c). Initial screening of hygromycin‐resistant transformants was conducted using diagnostic PCR with primer pairs MAT1‐2 FOR/MAT1‐2p2a, which hybridise outside and inside the replaced fragment, respectively, to identify putative homologous recombinants. PCR analysis identified a candidate transformant displaying amplification patterns consistent with successful *MAT1‐1* locus replacement (Figure S1b). Southern blot analysis of putative transformants confirmed the precise replacement of the 5 kb wild‐type BamHI fragment with a 7 kb fragment containing the hygromycin resistance cassette in one of the transformants (Figure S1c), designated *MAT1‐1*Δ. No ectopic integrations were detected in this strain, confirming the clean and complete deletion of the *MAT1‐1* locus.

We next generated complemented strains (*MAT1‐1*Δ + *MAT1‐1*) by reintroducing the complete *MAT1‐1* locus into the deletion mutant background via co‐transformation with a neomycin resistance cassette. Screening of neomycin‐resistant transformants was performed using diagnostic PCR with the primer pair MAT1‐1 COMPLFOR/MAT1‐1 COMPLREV, which amplify a 4.2 kb fragment spanning the entire *MAT1‐1* locus. PCR analysis confirmed successful reintroduction of the *MAT1‐1* locus in multiple transformants, as evidenced by amplification products matching the wild‐type 4287 strain pattern, whereas no amplification was observed in the *MAT1‐1*Δ deletion mutant or the *MAT1‐2*‐containing isolate MN25 used as negative controls (Figure S2).

Additionally, we created a set of mutants in which the *MAT1‐1* locus was replaced with either one or two copies of the *MAT1‐2* locus from isolate MN25. Transformant screening was conducted using a dual PCR approach: MAT1‐2 FORNEST/MAT1‐2 REVNEST primer pairs were used to confirm the presence of the inserted *MAT1‐2* locus (generating a 3.8 kb amplification product), whereas MAT1‐1 COMPLFOR/MAT1‐1 COMPLREV primers verified the absence of the endogenous *MAT1‐1* locus (no amplification expected). Four transformants displaying the expected PCR pattern, positive for *MAT1‐2* and negative for *MAT1‐1*, were selected for further analysis (Figure S3a). Southern blot analysis revealed that these transformants, designated *MAT1‐1*Δ + *MAT1‐2*, contained varying copy numbers of the inserted *MAT1‐2* locus (Figure S3b,c), with one harbouring a single‐copy integration while another contained two copies, providing an opportunity to examine dosage‐dependent effects of both *MAT* idiomorphs.

To generate strains carrying both mating‐type idiomorphs, isolate 4287 was transformed with the complete *MAT1‐2* locus from isolate MN25. PCR screening identified transformants containing both the endogenous *MAT1‐1* and the newly introduced *MAT1‐2* locus using diagnostic primer pairs MAT1‐2 FORNEST/MAT1‐2 REVNEST and MAT1‐5 FOR/MAT1‐3 REV to amplify the *MAT1‐2* and *MAT1‐1* loci, respectively. This dual PCR approach confirmed the presence of both idiomorphs through amplification of diagnostic fragments of 6.8 kb (*MAT1‐1*) and 3.8 kb (*MAT1‐2*) in selected transformants, whereas neither fragment was detected in the parental single‐idiomorph strains 4287 or MN25 (Figure S4a). Southern blot analysis of these strains designated *MAT1‐1* + *MAT1‐2* also showed that different transformants contained varying numbers of the inserted *MAT1‐2* locus (Figure S4b,c), allowing us to investigate the dose‐dependent effects of *MAT* idiomorphs.

To determine the influence of the genomic background on pseudo‐homothallic phenotypes, we performed the reciprocal transformation by introducing the complete *MAT1‐1* locus from 4287 into the MN25 isolate. PCR verification of the transformants with primers MAT1‐1 COMPLFOR/MAT1‐1 COMPLREV confirmed successful integration of the *MAT1‐1* locus (4.2 kb product). Multiple hygromycin‐resistant transformants displayed the presence of both idiomorphs in the MN25 genetic background (Figure S5a). Southern blot analysis of these *MAT1‐2* + *MAT1‐1* transformants revealed variable integration patterns, with some harbouring single‐copy insertions and others containing multiple copies of the introduced *MAT* locus (Figure S5b–d). Copy number determination was performed using idiomorph‐specific probes: a *MAT1‐1*‐specific probe constructed using MAT1‐1 SEQ1/MAT1‐1 COMPLREV primers and a *MAT1‐2*‐specific probe generated with MAT1‐2 SPLITFOR/MAT1‐2 SPLITREV primers.

### 2.2 *MAT* Loci Have Opposing Roles on Density‐Dependent Repression of Conidial Germination

To understand how *MAT* loci control fungal behaviour at the population level, we analysed the different strains for density‐dependent conidial germination, a critical process for infection initiation. At low inoculum concentration (LCI; 3.2 × 10^6^ conidia/mL), both wild‐type isolates 4287 (*MAT1‐1*) and MN25 (*MAT1‐2*) exhibited high germination rates between 80% and 93% (Figure 1b). By contrast, at high concentration of inoculum (HCI; 8.6 × 10^7^ conidia/mL), isolate 4287 displayed a low germination rate of 15% consistent with previous reports of density‐dependent suppression (Vitale et al. 2019), whereas isolate MN25 had a significantly higher rate of 68% (Figure 1c). This result suggests distinct regulatory functions of the two *MAT* idiomorphs in quorum sensing.

**FIGURE 2:**
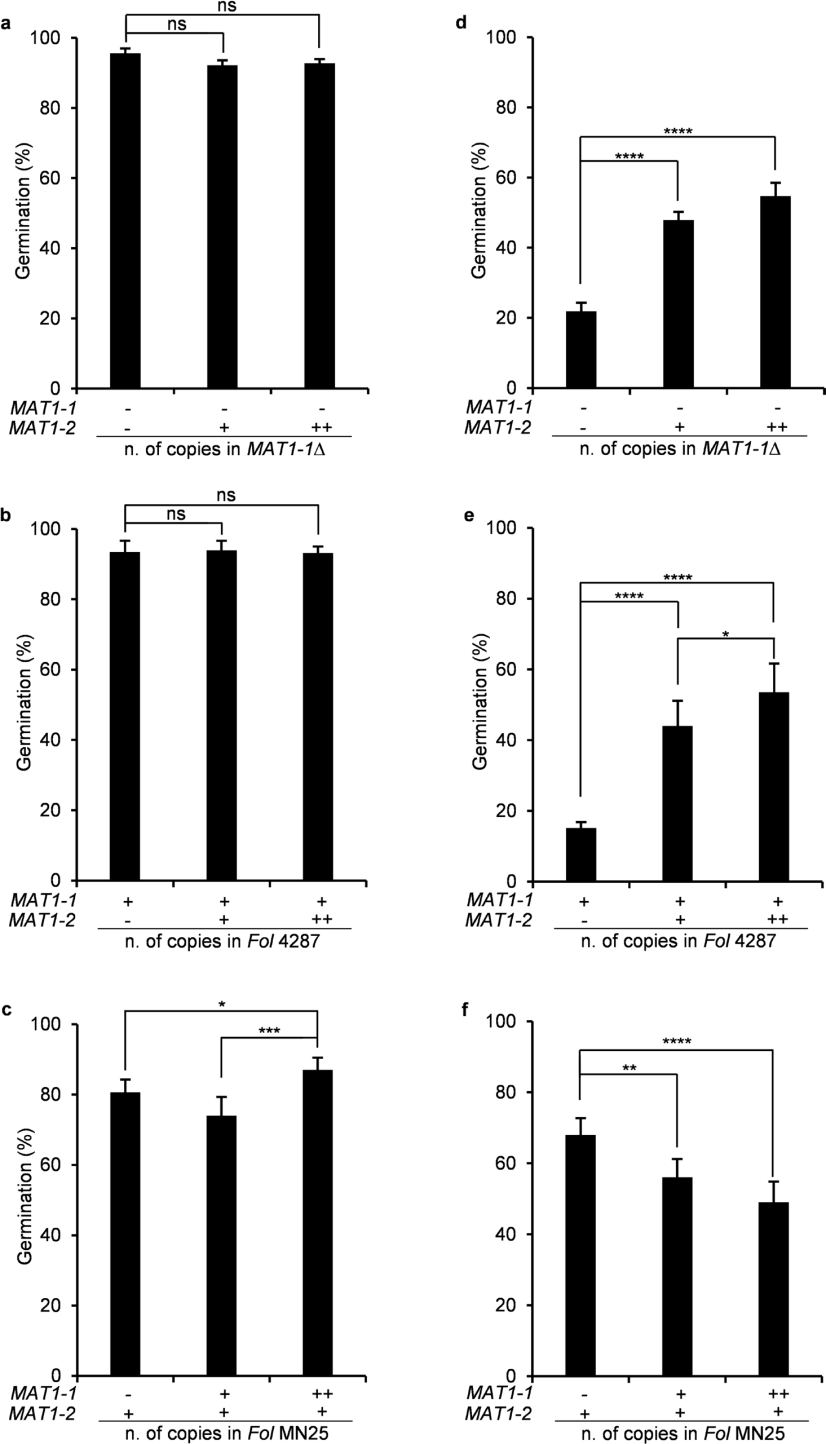
*MAT* loci exert dosage‐dependent control of density‐regulated germination. (a–c) Conidial germination at low concentration of inoculum (LCI; 3.2 × 10^6^ conidia/mL) showing similar high germination rates across the indicated strains of *Fusarium oxysporum* f. sp. *lycopersici*. Wild‐type isolate 4287 carries the *MAT1‐1* idiomorph, wild‐type isolate MN25 carries the *MAT1‐2* idiomorph. (d–f) Germination at high concentration of inoculum (HCI; 8.6 × 10^7^ conidia/mL) across the indicated strains revealing *MAT* locus‐dependent regulatory differences. Copy number effects are indicated for *MAT1‐1* and *MAT1‐2* insertions (− = absence, + = 1 copy, ++ = 2 copies), demonstrating progressive germination derepression or repression with increasing *MAT1‐2* or *MAT1‐1* dosage, respectively. Data represent means ± standard deviation from three independent experiments (*n* = 300 conidia per replicate). *p* values were calculated using Yates' corrected chi‐squared test (two‐sided). *p < 0.05; **p < 0.01; ***p < 0.001; ****p < 0.0001 vs. respective control strains; ns = not significant.

To establish causality, we systematically examined cell density‐dependent germination in the different *MAT* locus mutants. *MAT1‐1* deletion in isolate 4287 resulted in slight but significant derepression of conidial germination at HCI, with *MAT1‐1*Δ strains achieving 22% germination compared to 15% in wild‐type 4287 (*p* < 0.05) (Figure 1d). Importantly, this phenotype was fully restored in the complemented strains (*MAT1‐1*Δ + *MAT1‐1*), suggesting that *MAT1‐1* contributes to repression of conidial germination at high cell density. In line with this, insertion of the *MAT1‐1* locus into the MN25 background resulted in a copy number‐dependent reduction of the germination rate at HCI, confirming the inhibitory function of *MAT1‐1* across different genetic backgrounds (Figure 2e,f). On the other hand, introduction of *MAT1‐2* into a *MAT1‐1*Δ mutant (*MAT1‐1*Δ + *MAT1‐2*) or into the wild‐type 4287 (*MAT1‐1* + *MAT1‐2*) resulted in gene dosage‐dependent derepression of germination at HCI, with transformants carrying multiple *MAT1‐2* copies showing a stronger derepression (Figure 2a–d). Together, these results demonstrate that the two *MAT* idiomorphs function as opposing regulatory switches in quorum sensing‐dependent spore germination of *F. oxysporum*, with *MAT1‐1* repressing and *MAT1‐2* promoting germination at high cell density.

### 2.3 *MAT* Loci Modulate VHF in a Strain‐Dependent Manner

**FIGURE 3:**
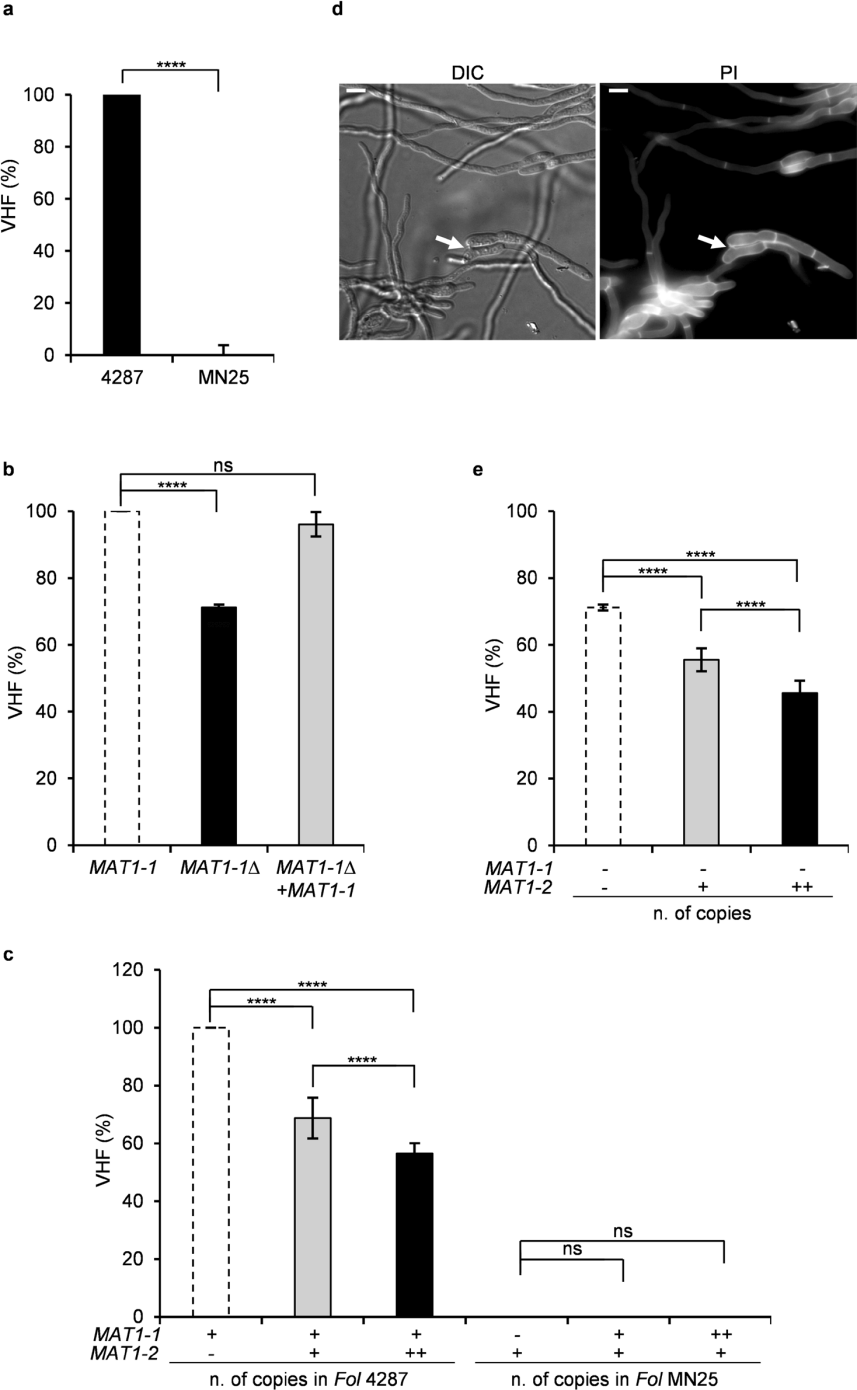
*MAT* loci differentially regulate vegetative hyphal fusion in *Fusarium oxysporum* f. sp. *lycopersici*. (a) Comparative analysis of vegetative hyphal fusion (VHF) efficiency between wild‐type isolates *MAT1‐1* (4287) and *MAT1‐2* (MN25). Fusion efficiency values are normalised to the *MAT1‐1* isolate, which was set to 100% for comparative analysis. ****p < 0.0001 vs. *MAT1‐1*. (b) VHF analysis of *MAT1‐1* deletion and complementation strains showing *MAT1‐1* requirement for efficient fusion. (c, e) Effect of *MAT1‐2* or *MAT1‐1* locus insertion on VHF efficiency. (c) *MAT1‐2* insertion in wild‐type 4287 background or *MAT1‐1* insertion in wild‐type MN25 background. (e) *MAT1‐2* insertion in *MAT1‐1*Δ background. Copy number is indicated (− = absence, + = 1 copy, ++ = 2 copies). Both panels show progressive reduction in VHF with increasing *MAT1‐2* copy number. All VHF assays were performed on minimal medium with 25 mM NaNO_3_ for 14 h at 28°C. Data represent mean ± standard deviation from three independent experiments (*n* = 300 germlings per experiment). Statistical significance: ****p < 0.0001 vs. respective control strains; ns = not significant. (d) Microscopic visualisation of rare hyphal fusion events in *MAT1‐2* (MN25) populations. Top panel: Differential interference contrast (DIC) imaging showing conidial anastomosis tube (CAT) formation between adjacent germlings. Bottom panel: Propidium iodide (PI) staining highlighting fusion points (arrows). Scale bar = 5 μm.

**FIGURE 4:**
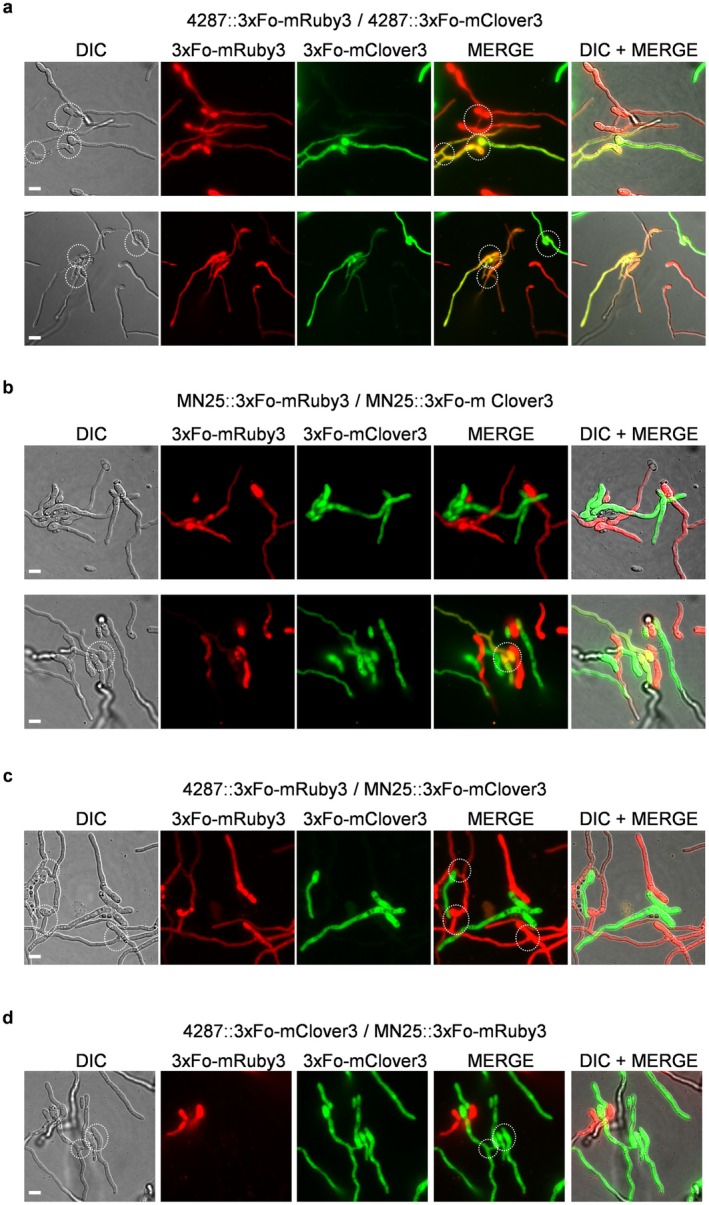
Fluorescence microscopy analysis of vegetative hyphal fusion (VHF) between differentially labelled *Fusarium oxysporum* f. sp. *lycopersici* isolates 4287 and MN25. (a–d) Representative images showing fusion assays between various *F. oxysporum* strain combinations expressing Fo‐mClover3 (green) or Fo‐mRuby3 (red) fluorescent proteins. Each panel displays differential interference contrast (DIC), individual fluorescence channels, and merged images. Successful VHF events are indicated by overlapping fluorescent signals in merged images, demonstrating absence/presence of cytoplasmic continuity between genetically identical or distinct fungal isolates. Scale bar = 5 μm.

We next investigated the role of *MAT* loci in VHF, a process resulting in multicellular development critical for pathogen fitness. The wild‐type *MAT1‐1 *isolate 4287 showed a hyphal fusion frequency of approximately 35% under standard assay conditions, which was linked to the presence of CATs (Figure 3a). Fluorescence microscopy with differentially labelled strains expressing either Fo‐mClover3 (green fluorescent protein) or Fo‐mRuby3 (red fluorescent protein) confirmed frequent cytoplasmic mixing between differentially labelled hyphae, resulting in cells co‐expressing both green and red fluorescent signals (Figure 4a). Targeted deletion of the entire *MAT1‐1* locus in isolate 4287 significantly reduced fusion efficiency (*p* < 0.0001), whereas genetic complementation of the mutant with the wild‐type *MAT1‐1* locus restored VHF levels, suggesting a role of *MAT1‐1* in promoting VHF (Figure 3b). On the other hand, introduction of either one or two copies of the *MAT1‐2* locus in the 4287 isolate (*MAT1‐1* + *MAT1‐2*) resulted in fusion frequency reductions of 32% or 44%, respectively compared to wild‐type (Figure 3c), whereas introduction of either one or two copies of the *MAT1‐2* locus in the *MAT1‐1*Δ mutant (*MAT1‐1*Δ + *MAT1‐2*) further reduced the VHF rate from 71% to 55% or 45% of that of the wild‐type strain (Figure 3e).

Strikingly, the isolate MN25 (*MAT1‐2*) exhibited an extremely low rate of hyphal fusion (< 1%). In line with this, co‐inoculation of differentially labelled MN25 germlings revealed only very rare events of cytoplasmic mixing, with the vast majority of germlings maintaining distinct single‐fluorophore expression patterns throughout the observation period (Figure 4b). Rare CAT formation events in MN25 populations were further confirmed by propidium iodide staining to assess cell wall continuity between adjacent fusing germlings (Figure 3d). Despite these sensitive detection methods, fusion events remained exceptionally rare in MN25 (Figure 3a). Most remarkably, when MN25 and 4287 strains expressing different fluorescent markers were co‐inoculated, only fusion events among 4287 germlings were detected, whereas no cytoplasmic mixing between MN25 and 4287 hyphae was observed despite physical proximity (Figure 4c,d). This observation demonstrates that fusion incompatibility between *MAT1‐1* and *MAT1‐2* isolates extends beyond simple frequency reduction. Our attempts to rescue fusion competence in the MN25 background through *MAT1‐1* insertion or co‐culture approaches with 4287 proved unsuccessful (Figures 3c and 4c,d). Fluorescence microscopy revealed no cytoplasmic mixing between MN25 and 4287 hyphae despite abundant fusion events among 4287 germlings in co‐culture conditions (Figure 4c,d). These findings indicate that MN25 probably represents a self‐incompatible strain carrying a mutation in a gene essential for hyphal fusion, suggesting that fusion deficiency in this isolate involves additional regulatory mechanisms beyond *MAT* locus composition alone.

### 2.4 *MAT* Loci Control Hyphal Aggregation

**FIGURE 5:**
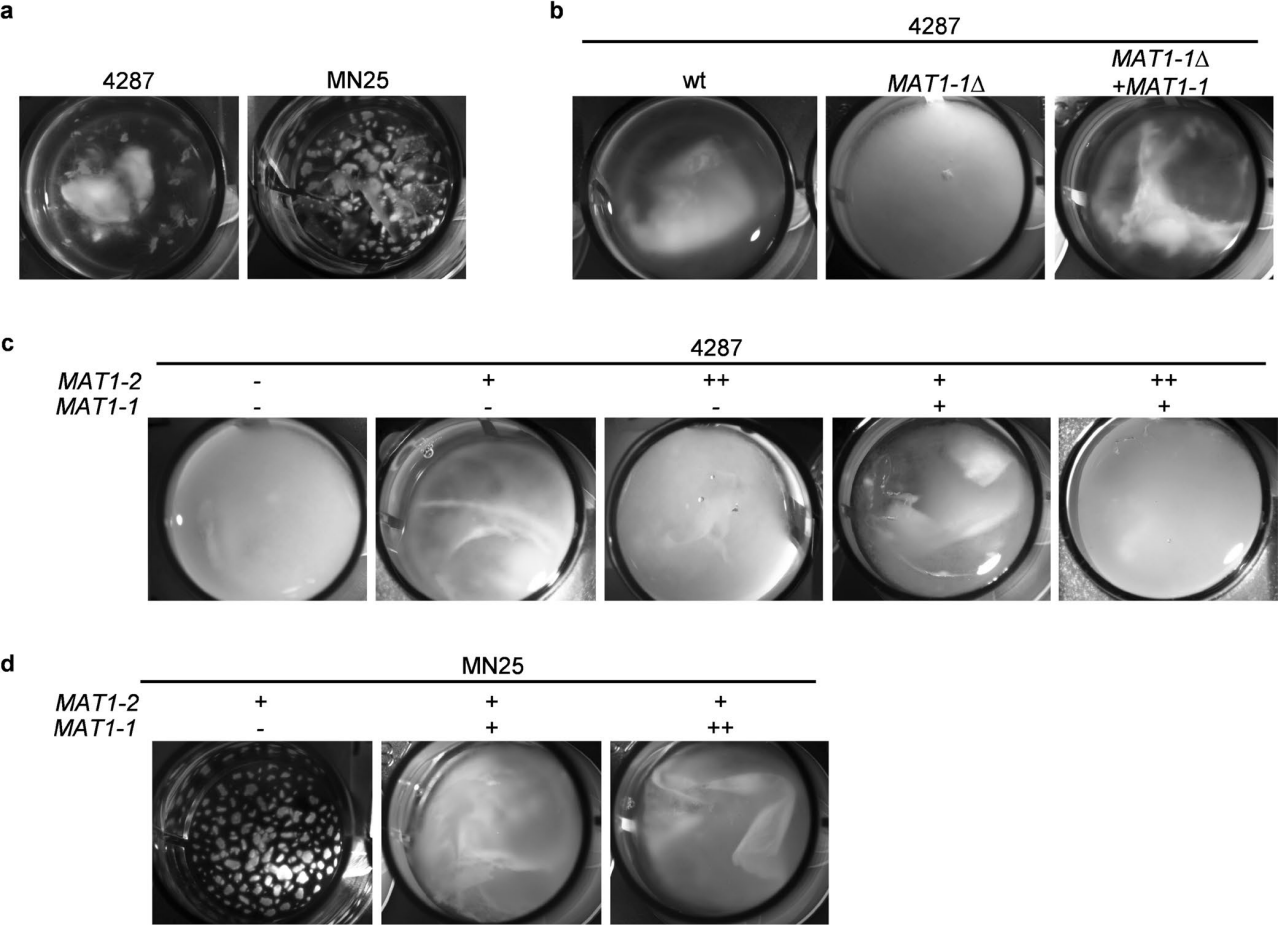
Differential effects of *MAT* loci on hyphal aggregation. (a) Morphological characterisation of mycelial aggregates formed by wild‐type *Fusarium oxysporum* f. sp. *lycopersici* isolates showing distinct architectures. Isolate 4287 forms large sheet‐like structures while MN25 produces smaller spherical aggregates. (b) Effect of *MAT1‐1* deletion on aggregate formation capacity in isolate 4287. Mycelia aggregation is completely abolished in *MAT1‐1*Δ deletion mutants, whereas fully restored, with a morphology resembling that of wild‐type 4287, in *MAT1‐1*Δ + *MAT1‐1* complementation mutants. (c) Dose‐dependent effects of *MAT* loci composition in isolate 4287 background. Single‐copy *MAT1‐2* insertion (+) or combination with single‐copy *MAT1‐1* (+) results in mild aggregation phenotypes, whereas double‐copy *MAT1‐2* insertions (++) or absence of *MAT* loci (−) completely abolish aggregate formation. (d) *MAT* loci dosage effects in MN25 background showing altered aggregation patterns. Single‐copy *MAT1‐1* insertion (+) modifies aggregate architecture compared to wild‐type, whereas double‐copy insertion (++) completely reduces aggregate formation, indicating disrupted *MAT* loci balance or critical dosage thresholds. Representative images from three independent experiments are shown. Cultures were grown for 48 h at 28°C with orbital shaking at 170 rpm before microscopic analysis.

Analysis of hyphal aggregate formation was conducted, because this process contributes to surface adhesion and biofilm‐like structures that enhance pathogen survival (Di Pietro et al. 2001; Falanga et al. 2022). We found that both wild‐type isolates formed visible aggregates in liquid culture, which displayed clear morphological differences (Figure 5a): while 4287 produced large, sheet‐like structures with extensive hyphal networks, probably as a result of hyphal adhesion and fusion, MN25 only formed small, compact spherical aggregates, probably due to the absence of VHF.

Importantly, *MAT1‐1* deletion in 4287 completely abolished aggregate formation, resulting in dispersed hyphal growth and failure to organise into multicellular structures (Figure 5b). Genetic complementation fully restored aggregation capacity in the *MAT1‐1*Δ + *MAT1‐1* strain, confirming the essential role of *MAT1‐1* in this developmental process. The correlation between reduced fusion efficiency and impaired aggregation in the *MAT1‐1*Δ mutant suggests that cell–cell fusion promotes aggregate formation.

Systematic genetic dissection of *MAT* loci dosage and composition revealed dose‐dependent regulatory mechanisms governing aggregation control (Figure 5c,d). In the 4287 background, absence of either *MAT* locus individually (−) completely prevented aggregate formation. Single‐copy insertions of *MAT1‐2* (+) or *MAT1‐1* (+) produced mild aggregation phenotypes with reduced structural organisation compared to wild‐type. Double‐copy insertions (++) of either locus also abolished aggregation, indicating critical dosage requirements for optimal function.

Analysis of the MN25 background demonstrated strain‐specific regulatory differences (Figure 5d). Single‐copy *MAT1‐1* insertion in the MN25 background reduced aggregate formation compared to the wild‐type MN25, resulting in increased culture turbidity, whereas double‐copy *MAT1‐1* insertion further impaired aggregate formation. This indicates that *MAT1‐1* has differential roles in aggregation, which are likely dependent or independent of hyphal fusion.

### 2.5 *MAT* Loci Transcriptionally Regulate Autocrine Pheromone Signalling Genes

To study the role of *MAT* loci in APS, reverse transcription‐quantitative real‐time PCR (RT‐qPCR) analysis was performed to measure transcript levels of pheromone/receptor pair genes at high cell density. Initial characterisation of wild‐type isolates demonstrated that both 4287 (*MAT1‐1*) and MN25 (*MAT1‐2*) strains co‐expressed both pheromone precursor/receptor pairs (*MFα*/*ste2* and *MFa*/*ste3*), contradicting canonical heterothallic expression patterns observed in other ascomycetes (Wang et al. 2012) (Figure S6). This atypical expression profile had been previously documented for isolate 4287 by Vitale et al. (2019), and our findings extend this observation to the *MAT1‐2* isolate MN25. Notably, the *MAT1‐2* isolate exhibited significantly higher transcript levels of *MFα*, *MFa* and *ste3* genes compared to the *MAT1‐1* strain, whereas *ste2* levels were similar between these strains (Figure S6a,b). Furthermore, expression of the *Bar1* protease, which specifically cleaves and inactivates α‐pheromone (Hicks and Herskowitz 1976), was 10‐fold higher in the *MAT1‐2* strain than in the *MAT1‐1* isolate (Figure S6c).

**FIGURE 6:**
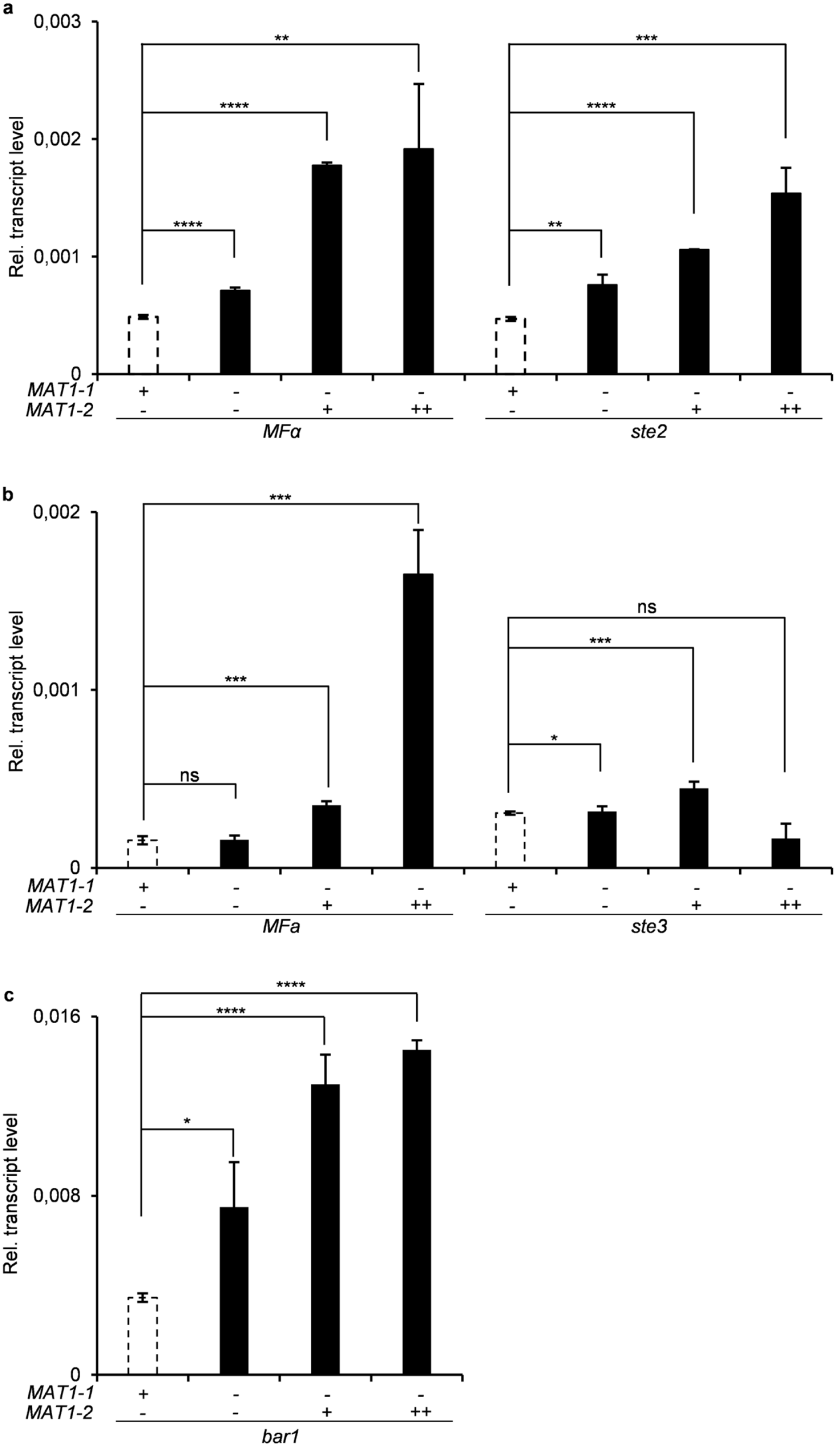
Opposing regulatory roles of *MAT1‐1* and *MAT1‐2* loci in autocrine pheromone signalling gene expression. Reverse transcription‐quantitative real‐time PCR (RT‐qPCR) analysis demonstrating dose‐dependent effects of *MAT* loci on APS‐related gene transcription in the *Fusarium oxysporum* f. sp. *lycopersici* isolate 4287. Copy number is indicated (− = absence, + = 1 copy, ++ = 2 copies). *MFα*, *ste2*, *bar1*, *MFa* and *ste3* genes were measured by RT‐qPCR analysis of cDNA obtained from *F. oxysporum* germlings grown in germination minimal medium at high cell density. Transcript levels were calculated using the ΔΔ*C*_t_ method and normalised to the peptidyl‐prolyl isomerase (*ppi*) reference gene. Bars represent means ± SE from three independent experiments with three technical replicates each. Statistical significance was assessed by Welch's *t*‐test (two‐tailed) (*p < 0.05; **p < 0.01; ***p < 0.001; ****p < 0.0001 vs. respective control strains; ns = not significant).

*MAT1‐1* deletion in 4287 resulted in a slight but significant upregulation of the *MFα*, *ste2* and *bar1* genes, but had no or only marginal effects on a‐pheromone‐related gene expression (*MFa*, *ste3*), suggesting that *MAT1‐1* contributes to transcriptional repression of the α‐pheromone signalling pathway genes (Figure 6). Notably, insertion of one or two *MAT1‐2* copies into the *MAT1‐1*Δ background resulted in further dosage‐dependent upregulation of *MFα*, *ste2*, *MFa* and *bar1* (Figure 6). These findings suggest that *MAT1‐2* functions as a positive regulator of APS‐related transcription, indicating antagonistic regulatory roles of the two mating‐type loci.

Analysis of *MAT* loci interactions within intact genetic backgrounds revealed sophisticated compensatory mechanisms governing pheromone pathway homeostasis. In the 4287 background, single‐copy *MAT1‐2* insertion enhanced both pheromone genes and *ste2* receptor expression while paradoxically reducing *bar1* levels, suggesting pathway‐specific feedback regulation (Figure S7). Double‐copy insertion amplified *MFα* transcript levels but normalised receptor and protease expression near wild‐type levels, indicating the existence of dosage‐sensitive regulatory checkpoints that prevent excessive signalling (Figure S7). Conversely, *MAT1‐1* insertion into the MN25 background produced biphasic dose‐responses, with single‐copy insertion dramatically elevating most APS components (particularly *bar1*, reaching ~9‐fold induction) while double‐copy insertion reduced transcript levels below single‐copy conditions (Figure S8). This non‐linear response pattern suggests threshold‐dependent regulatory mechanisms or competitive inhibition phenomena that become activated at higher *MAT1‐1* concentrations.

### 2.6 *MAT1‐1* Contributes to Virulence on Tomato Plants

**FIGURE 7:**
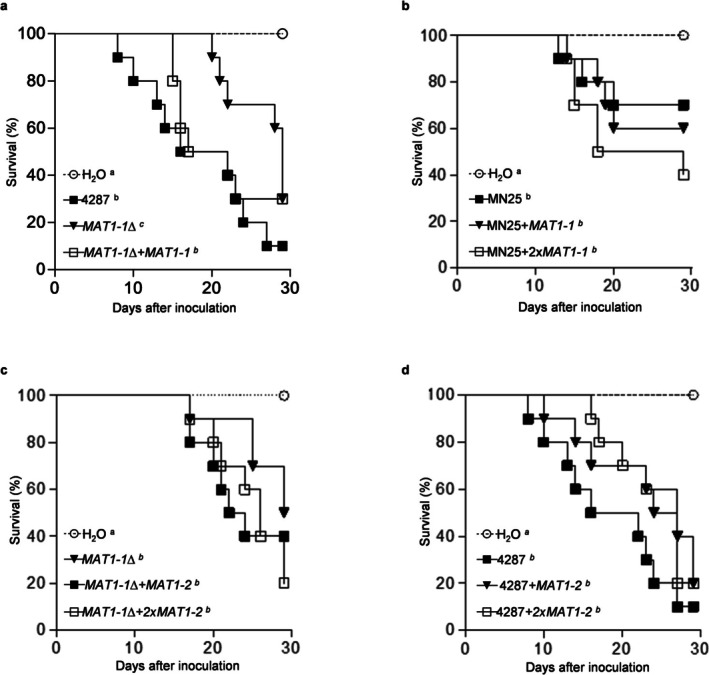
*MAT* loci exhibit differential contributions to fungal virulence through isolate‐specific regulatory mechanisms. Kaplan–Meier survival analysis of 14‐day‐old tomato seedlings ( *Solanum lycopersicum* 'Monika') following root‐dip inoculation (5 × 10^6^ conidia/mL). (a) *MAT1‐1* deletion significantly reduces pathogenicity (*p* = 0.026, log‐rank test) while genetic complementation restores virulence. (b) *MAT1‐1* insertion into MN25 background fails to enhance virulence regardless of copy number, indicating requirement for strain‐specific cofactors. (c) *MAT1‐2* insertion into *MAT1‐1*Δ background shows non‐significant trends toward delayed infection kinetics. (d) *MAT1‐2* insertion into wild‐type 4287 produces modest virulence attenuation trends. Each survival curve represents *n* = 15 plants per treatment across three independent experiments. Statistical comparisons by log‐rank (Mantel–Cox) test. Different letters in the legend indicate significant survival differences.

To assess *MAT* loci contributions to virulence while accounting for race‐specific differences between 4287 (race 2) and MN25 (race 3), we employed reciprocal insertion experiments that enable separating *MAT*‐specific effects from background‐dependent pathogenicity levels. Infection assays on tomato seedlings ( *Solanum lycopersicum* 'Monika') by root‐dip inoculation demonstrated distinct virulence levels of the two wild‐type isolates, with 4287 causing higher mortality than MN25 (Figure 7a,b). Targeted deletion of the *MAT1‐1* locus in 4287 resulted in significantly reduced pathogenicity (*p* = 0.026), with infected plants showing longer survival times (Figure 7a), whereas complementation of the mutant (*MAT1‐1*Δ + *MAT1‐1*) largely restored wild‐type virulence (*p* = 0.35). Reciprocal insertion experiments revealed background‐dependent regulatory mechanisms. Thus, introduction of *MAT1‐1* into the MN25 genetic background failed to significantly enhance virulence regardless of copy number, suggesting that virulence enhancement requires additional strain‐specific cofactors or regulatory networks absent in the MN25 lineage (Figure 7b). Analysis of *MAT1‐2* insertional mutants revealed a slight, but consistent trend toward reduced virulence across multiple genetic contexts. In the *MAT1‐1*Δ background, neither single‐copy (*p* = 0.34) nor double‐copy (*p* = 0.15) *MAT1‐2* insertion significantly altered pathogenicity, although both conditions exhibited delayed infection kinetics (Figure 7c). Similarly, *MAT1‐2* insertion into the wild‐type 4287 isolate produced a non‐significant but consistent attenuation of pathogenicity (single copy: *p* = 0.07; double copy: *p* = 0.12) (Figure 7d). Taken together, these findings suggest that mating type influences virulence quantitatively rather than affecting infection per se, with *MAT1‐1* enhancing virulence in the 4287 background, and *MAT1‐2* exhibiting a trend toward slightly reduced virulence.

## 3 Discussion

In the current study, we demonstrate that the plant pathogen *F. oxysporum* has co‐opted *MAT* loci and APS to regulate functions unrelated to mating such as quorum sensing, developmental coordination and pathogenicity. The finding that *F. oxysporum*, which lacks a known sexual cycle, co‐expresses both pheromone‐receptor pairs contradicts the traditional paradigm that fungal pheromones function exclusively in sexual reproduction (Alvaro and Thorner 2016; Johnson 2003), representing a remarkable example of evolutionary repurposing extending beyond simple gene retention in asexual lineages. We further show that the two different *MAT* loci have opposing regulatory roles: *MAT1‐1* functions mainly in transcriptional repression of APS while *MAT1‐2* promotes its derepression. This duality creates a bistable switch for population‐level coordination of critical developmental decisions. Our results are in line with emerging evidence from other fungal systems suggesting that *MAT* loci might carry out additional functions unrelated to sexual reproduction, even when the sexual reproduction machinery is retained (Fraser et al. 2004; Nieuwenhuis et al. 2013; Wang et al. 2017). These opposing regulatory roles of *MAT1‐1* and *MAT1‐2* are mediated through differential expression of key downstream effectors. The secreted protease Bar1, which specifically cleaves the α‐pheromone peptide to reduce its bioavailability, has been suggested to modulate the balance between germination inhibition by α‐pheromone/Ste2 and promotion by a‐pheromone/Ste3 (Jones et al. 2015; Vitale et al. 2019). The 10‐fold higher transcript levels of *bar1* detected in the *MAT1‐2* isolate MN25 compared to *MAT1‐1* isolate 4287 provide a possible explanation for the increased ability of this strain to germinate at high cell density. Elevated Bar1 activity would prevent accumulation of α‐pheromone to inhibitory concentrations, allowing the a‐pheromone/Ste3 module to shift the signalling equilibrium toward derepression. This regulatory model mirrors the barrier activity mechanism documented in *Candida albicans* , where Bar1‐mediated pheromone degradation determines whether sexual reproduction proceeds via homothallic or heterothallic pathways (Alby et al. 2009). In *C. albicans* , *bar1* deletion enables autocrine mating responses within an unisexual population by allowing α‐pheromone to accumulate and activate same‐sex mating via the Ste2 receptor. Similarly, in *Saccharomyces cerevisiae* , Bar1 increases pheromone gradients to facilitate partner discrimination during mating, suggesting that this protease has a regulatory rather than a purely degradative function across different fungal lineages (Barkai et al. 1998).

In strains carrying two copies of *MAT1‐2*, we observed transcriptional upregulation of *bar1* coupled with *ste3* downregulation. This suggests a homeostatic mechanism maintaining signalling balance between the two pheromone systems, as previously described in yeast (Bardwell 2004). Recent evidence from mushroom‐forming basidiomycetes has demonstrated dosage compensation mechanisms at *MAT* loci: HD (homeodomain transcription factor genes) and P/R (pheromone and pheromone receptor) genes show differential expression levels between monokaryons and dikaryons, specifically to compensate for *ste3* pheromone receptor expression (Wang et al. 2021). In the MN25 background we observed high *bar1* upregulation upon single copy *MAT1‐1* insertion but reduced *bar1* expression at double copy insertion, suggesting a threshold‐dependent regulatory switch or a competitive inhibition mechanism. These non‐linear dynamics indicate the presence of an optimal *MAT* locus stoichiometry, highlighting evolutionary fine‐tuning of these ancient signalling modules. This concept of dosage‐dependent regulation is further substantiated by examining the functional consequences of Bar1 modulation. The correlation between *bar1* expression levels and germination phenotypes across all genetic backgrounds provides compelling evidence that this protease has a pivotal role in translating *MAT* locus input into developmental outputs. Indeed, in genomic setups where *bar1* expression was elevated (MN25, *MAT1‐1*Δ and *MAT1‐2* insertion strains), inhibition of spore germination at high cell density was partially abolished, whereas in strains with low *bar1* transcript levels (4287, *MAT1‐1*Δ + *MAT1‐1*), density‐dependent inhibition remained active. This suggests that Bar1 acts as a critical control point for phenotypic outcomes, analogous to key developmental switches in multicellular organisms (Chang et al. 2009). Bar1 is conserved across phylogenetically distant fungi including *Saccharomyces*, *Candida* and *Fusarium* pointing toward an ancient regulatory role co‐opted for diverse signalling processes, rather than mere degradative function (Barkai et al. 1998; Jones et al. 2015; Vitale et al. 2019). The capacity of Bar1 to create a signalling gradient and to prevent autocrine feedback has likely facilitated the evolutionary transition from paracrine sexual to autocrine developmental signalling (Doğaner et al. 2016). Bar1 and the pheromone signalling‐related genes thus exemplify mating system repurposing: from suppressing autocrine mating signals to regulating density‐dependent germination in asexual pathogens (Vitale et al. 2019). Beyond developmental regulation, our data indicate that the pheromone signalling network also interfaces with pathogenicity determinants. Loss of *MAT1‐1* in isolate 4287 led to significantly reduced virulence suggesting a role of this locus in plant infection. These pathogenicity effects likely reflect interactions of *MAT* loci with strain‐specific virulence networks rather than a direct role in infection, as evidenced by the background‐dependent responses observed in the 4287 and MN25 isolates. Similar results were reported in *Villosiclava virens*, where *MAT1‐1‐3* deletion reduced pathogenicity on rice (Yong et al. 2020) and in *Fusarium graminearum*, where *MAT* genes showed host‐specific virulence contributions (Zheng et al. 2013). This suggests that APS networks coordinate developmental decisions while modulating pathogenic capacity according to cell density, consistent with reports showing that quorum sensing regulates virulence factors in fungal pathogens (Albuquerque et al. 2014; Tian et al. 2021). A molecular link between these observations emerged from recent work connecting pheromone signalling to secondary metabolite production. Critically, Staropoli et al. (2024) demonstrated that pheromone signalling represses fusaric acid production in *F. oxysporum*, establishing a direct mechanistic link between APS and virulence factor regulation. Fusaric acid (FA) is a phytotoxin that contributes significantly to *F. oxysporum* virulence on both plant and mammalian hosts through metal ion chelation and disruption of cellular homeostasis (López‐Díaz et al. 2018). Recent work demonstrates that virulence‐promoting conditions such as alkaline pH, low iron availability, and activation of the cell wall integrity MAPK pathway enhance FA production (Palmieri et al. 2023), suggesting a coordination between environmental sensing, density‐dependent signalling, and virulence factor deployment. FA production also mediates broader ecological interactions by influencing rhizosphere microbiota assembly, potentially affecting disease suppression (Jin et al. 2024). In addition, the pheromone receptor Ste2 mediates chemotropism toward host‐derived signals (Sridhar et al. 2020; Turrà et al. 2015), further supporting the evolutionary co‐option of the pheromone signalling modules. The strain‐specific role of *MAT* loci in infection indicates that additional genetic components control pathogenicity. These include core effectors, lineage‐specific chromosomes and multiple regulatory networks (Redkar et al. 2022; Zuriegat et al. 2021). *MAT1‐1* may thus link quorum‐sensing inputs with virulence factor expression, enabling infection strategy modulation based on population density. The minor virulence effects observed in our study suggest that *MAT* loci function as regulatory modulators rather than as essential infection determinants. Collectively, these findings position *F. oxysporum* as a paradigmatic system for understanding how conserved sexual regulatory machinery can be evolutionarily repurposed to govern complex behaviours in asexual pathogens. The modulatory effect on virulence further suggests that *MAT* loci operate within sophisticated regulatory hierarchies where multiple genetic components collectively determine the infection outcome. Such network‐level organisation suggests that evolutionary repurposing of the sexual machinery creates regulatory flexibility rather than a rigid developmental switch.

In summary, our findings suggest that sexual regulatory networks provide robust scaffolds for signalling innovation in asexual lineages (Nieuwenhuis and James 2016). Pheromone signalling systems have been repurposed for pathogen–host interactions across diverse *Fusarium* species: in *F. oxysporum*, the α‐pheromone receptor Ste2 mediates both autocrine population density sensing and chemotropic sensing of plant signals (Turrà et al. 2015; Vitale et al. 2019), whereas in *Fusarium circinatum*, Ste2 orchestrates chemotropic growth toward pine root extracts (Ramaswe et al. 2024). These signalling networks critically impact quorum sensing, germination control and host recognition, directly affecting pathogen fitness and disease establishment. Comparative transcriptomic analysis between wild‐type and *MAT* deletion mutants represents a future direction for identifying *MAT* loci target genes beyond the known pheromone signalling components characterised here, potentially revealing connections to secondary metabolite production, virulence factor networks, and broader developmental programs.

The mechanistic insights into the pheromone‐processing protease Bar1 offer new opportunities for therapeutic intervention. Recent work linking pheromone signalling to FA production (Staropoli et al. 2024) suggests that targeting these signalling pathways could provide novel pathogen control strategies. Such approaches represent alternatives to traditional antifungals where resistance evolution remains a critical challenge (Perfect 2017). Our study establishes *F. oxysporum* as a paradigmatic example of sexual regulatory machinery repurposing, demonstrating that *MAT* loci function as master regulators of density‐dependent developmental switching in one of agriculture's most devastating pathogen complexes (Dean et al. 2012).

## 4 Experimental Procedures

### 4.1 Fungal Strains and Cultivation Requirements

Wild‐type isolates of *F. oxysporum* f. sp. *lycopersici* were obtained from the Fungal Genetics Stock Center (https://www.fgsc.net/about.html#intro) including isolate 4287 (NRRL 34936, *MAT1‐1*) and isolate MN25 (NRRL 54003, *MAT1‐2*). The derived mutant strains used in this study are detailed in Table 1. All strains were maintained as microconidial suspensions in 30% glycerol at −80°C for long‐term storage, following established protocols (Di Pietro and Roncero 1998).

**TABLE 1:** *Fusarium oxysporum* f.sp. *lycopersici* strains used in this study.

Strain name	FOXG/FOWG	Genetic background	Genotype	Resistance	References
4287 (*MAT1‐1*)	—	NRRL 34936	Wild type Race 2	None	Fungal Genetics Stock Center
MN25 (*MAT1‐2*)	—	NRRL 54003	Wild type Race 3	None	Fungal Genetics Stock Center
*MAT1‐1∆*	FOXG_03616; FOXG_03615; FOXG_17746	NRRL 34936	Knockout mutant of the entire *MAT1‐1* locus	Hyg^R^	This study
*MAT1‐1∆* + *MAT1‐1*	FOXG_03616; FOXG_03615; FOXG_17746	NRRL 34936	Complemented strain of the entire *MAT1‐1*Δ locus	Hyg^R^::Neo^R^	This study
*MAT1‐1∆* + *MAT1‐2*	FOWG_12730; FOWG_12731	NRRL 34936	Insertional mutant obtained by inserting the *MAT1‐2* locus (containing one copy or two copy inserted)	Hyg^R^::Neo^R^	This study
*MAT1‐1* + *MAT1‐2*	FOWG_12730; FOWG_12731	NRRL 34936	Insertional mutant obtained by inserting the *MAT1‐2* locus (one or two copies inserted)	Hyg^R^	This study
*MAT1‐2* + *MAT1‐1*	FOXG_03616; FOXG_03615; FOXG_17746	NRRL 54003	Insertional mutant obtained by inserting the *MAT1‐1* locus (one or two copies inserted)	Hyg^R^	This study
*Fol4287‐mClover3*			*Fol4287::An‐gpdaP:3×Fo mClover3*:3×FLAG::*HYG*	Hyg^R^	Redkar et al. (2022)
*Fol4287‐mRuby3*		NRRL 34936	*Fol4287::An‐gpdaP:3×Fo mRuby3*:3×FLAG::*HYG*	Hyg^R^	This study
*FolMN25‐mClover3*		NRRL 54003	*FolMN25::An‐gpdaP:3×Fo mClover3*:3×FLAG::*HYG*	Hyg^R^	Redkar et al. (2022)
*FolMN25‐mRuby3*		NRRL 54003	*FolMN25::An‐gpdaP:3×Fo mRuby3*:3×FLAG::*HYG*	Hyg^R^	This study

For routine propagation and DNA isolation, cultures were grown in potato dextrose broth (PDB) at 28°C with continuous shaking at 170 rpm for 3–5 days. Transformant selection was performed using potato dextrose agar (PDA) or PDB supplemented with either hygromycin B (55 μg/mL) or G418 (100 μg/mL), depending on the resistance marker employed.

### 4.2 Generation of *MAT* Locus Mutants

PCR amplifications were routinely performed using the Expand High Fidelity PCR System (Roche) on a MJ Mini personal thermal cycler (Bio‐Rad) with standard cycling conditions and primer‐specific annealing temperatures calculated using the Oligo 7.0 software. All primer sequences are listed in Table 2. Protoplast‐mediated transformation of fungal strains was performed using protoplasts prepared following Di Pietro et al. (2001) with modifications, and transformants were subsequently purified through monoconidial isolation as described previously (Di Pietro and Roncero 1998). For generation of *MAT1‐1* deletion mutants, targeted replacement of the entire genomic locus was achieved using the split‐marker strategy (Catlett et al. 2003; Rispail and Di Pietro 2009) following the scheme represented in Figure S1. The hygromycin B resistance cassette (Hyg^R^) from pAN7‐1, under the control of the *Aspergillus nidulans gpdA* promoter and the *trpC* terminator (Punt et al. 1987), served as the selection marker. Flanking regions of the *MAT1‐1* locus (1150 bp upstream and 1436 bp downstream) were PCR‐amplified and fused with partially overlapping truncated hygromycin resistance cassette fragments using fusion PCR methodology (Yang et al. 2004), and the resulting split‐marker constructs were used directly for protoplast‐mediated transformation. Complementation strains were generated by co‐transformation of *MAT1‐1*Δ protoplasts with the complete *MAT1‐1* locus (4.2 kb, amplified using primers MAT1‐1 COMPLFOR/MAT1‐1 COMPLREV) and the neomycin resistance cassette (2.7 kb, amplified using primers GPDA15B/TRPTER8B) containing the *NPTII* gene under transcriptional control of the *A. nidulans gpdA* promoter and *trpC* terminator (Palos‐Fernández et al. 2022).

**TABLE 2:** Synthetic oligonucleotides used in this study.

Primer name	Sequence (5′–3′)	Use
MAT 1‐5 FOR	ATGACTACACACCCACCCAC	Knockout *MAT1‐1* locus
MAT 1‐5 REV	TTTACCCAGAATGCACAGGTACACTTGTTTAATGGCGTAAAACAATGTCAAGG	Knockout *MAT1‐1* locus
MAT 1‐3 FOR	TGGTCGTTGTAGGGGCTGTATTAGGTCTCGAGAAATAGCCAAACCTCTCTCTG	Knockout *MAT1‐1* locus
MAT 1‐3 REV	TGATACCAGAATTTGACCGCC	Knockout *MAT1‐1* locus
MAT 1‐5 FORNEST	TCTCTCCAAGCTACCTCCCT	Knockout *MAT1‐1* locus
MAT 1‐3 REVNEST	GCCGACGAGGACAGACACA	Knockout *MAT1‐1* locus
HYG‐G	CGTTGCAAGACCTGCCTGAA	Hyg^R^ cassette
HYG‐Y	GGATACCTCCGCTCGAAGTA	Hyg^R^ cassette
GPDA15B	CGAGACCTAATACAGCCCCT	Hyg^R^ cassette/Neo^R^ cassette
TRPTER15B	GGATCCAAACAAGTGTACCTGTGCATTC	Hyg^R^ cassette/Neo^R^ cassette
MAT 1‐1 COMPLFOR	CAGAGAGAGGTTTGGCTATTTC	*MAT1‐1* locus reinsertion
MAT1‐1 COMPLREV	CCTTGACATTGTTTTACGCCAT	*MAT1‐1* locus reinsertion/*MAT1‐1* probe
MAT 1‐2 FORNEST	ATCCACAAATACATCGCTTCCT	*MAT1‐2* locus insertion/screening of *MAT1‐2* insertional transformants
MAT1‐2 REVNEST	AGGAACACAGAAGATTGGCAC	*MAT1‐2* locus insertion/screening of *MAT1‐2* insertional transformants
MAT1‐1 SEQ1	GGTAGTCAAATCAATGCGGTC	*MAT1‐1* probe
MAT1‐2 SPLITFOR	TTTGGTGTTGTGAGGAGGAGA	*MAT1‐2* probe
MAT1‐2 SPLITREV	TGCTAGATTGACTGTGGTGGT	*MAT1‐2* probe
MAT1‐2p2A	ATAAAGGGCTGAAGCTGAACGAG	Screening of *MAT1‐1*∆ transformants
MAT1‐2 FOR	CCTCTGTCGTTGCTTCCACC	Screening of *MAT1‐1*∆ transformants
α‐FOR	AACGCCCTCCACGCAACT	Real‐time qPCR primer (*MFα*)
α‐REV	AGCATCGGGGAAGAAGGTTT	Real‐time qPCR primer (*MFα*)
A‐FOR	CCTTCCACCAAGAACACCAC	Real‐time qPCR primer (*MFa*)
A‐REV2	AGGGGGTAGCCGGGGGTTT	Real‐time qPCR primer (*MFa*)
STE2‐FOR2	ACATCACGTTCTTAGCAGCAG	Real time qPCR primer (*ste2*)
STE2‐REV2	TACACGAGCAGATGAAGAGAC	Real‐time qPCR primer (*ste2*)
STE3‐FOR	TATGGTCTTCTTCGTCCTGG	Real‐time qPCR primer (*ste3*)
STE3‐REV	CAAAGGGCTGAAGAGGAAATG	Real‐time qPCR primer (*ste3*)
BAR1‐FOR	AAGAGAGACGGGACAATAGAC	Real‐time qPCR primer (*bar1*)
BAR1‐REV	AAGAGAGACGGGACAATAGAC	Real‐time qPCR primer (*bar1*)
AR17_qP_ppi_Fol‐F	AAGGGTGACCAGTTCGATAG	Real‐time qPCR primer (*ppi*)
AR17_qP_ppi_Fol‐R	TTCTCGCCGAGCTTCATTTG	Real‐time qPCR primer (*ppi*)

Insertional mutants containing both *MAT* idiomorphs were generated by co‐transformation with the complete heterologous *MAT* locus and appropriate selection cassettes. For 4287 background strains, the *MAT1‐2* locus (3.8 kb) was amplified from MN25 genomic DNA using primers MAT1‐2 FORNEST/MAT1‐2 REVNEST and co‐transformed with the Hyg^R^ resistance cassette (2.7 kb) from the pAN7‐1 plasmid, amplified using primers GPDA15B/TRPTER15B. For MN25 background strains, the *MAT1‐1* locus (4.2 kb) was amplified from 4287 genomic DNA using primers MAT1‐1 COMPLFOR/MAT1‐1 COMPLREV and co‐transformed with the same Hyg^R^ cassette. Additionally, *MAT1‐1*Δ insertional mutants containing the heterologous *MAT1‐2* locus (*MAT1‐1*Δ + *MAT1‐2*) were obtained by co‐transforming *MAT1‐1*Δ protoplasts with the same *MAT1‐2* amplicon described above and the Neo^R^ cassette as selective marker. Co‐transformation ratios were optimised at 2–7 μg insert DNA to 1–3 μg selection cassette. All transformants were confirmed by PCR screening, Southern blot analysis, or both techniques (Figures [Link], [Link], [Link], [Link], [Link]).

### 4.3 Generation of Fluorescently Labelled Fungal Transformants

Plasmid constructs carrying *F. oxysporum* codon‐optimised *mClover3* and *mRuby3* fluorescent protein coding genes were used for generating cytoplasmic fluorescence‐tagged fungal strains. The Fo‐mRuby3 expression cassette was obtained using the same methodology as previously described for Fo‐mClover3 construct generation (Redkar et al. 2022), with three copies of Fo‐mRuby3 replacing the three copies of Fo‐mClover3 coding sequence while maintaining the same plasmid backbone, codon optimisation parameters and regulatory elements.

Fluorescently‐labelled strains of *F. oxysporum* f. sp. *lycopersici* MN25 (expressing either Fo‐mClover3 or Fo‐mRuby3) and 4287 (expressing Fo‐mRuby3) were obtained by co‐transforming fungal protoplasts with the respective fluorescent protein expression cassettes and a hygromycin resistance cassette amplified from plasmid pAN7‐1 following the above‐described protocols. A 4287 strain expressing three copies of Fo‐mClover3 had been previously generated and characterised (Redkar et al. 2022, 2023).

Cytoplasmic fluorescent protein expression was observed and quantified in at least 20 independent transformants using an Axio Imager M2 microscope (Zeiss) equipped with an Evolve Photometrics EM512 digital camera and appropriate filter sets (GFP filter set: BP 450/490, FT 510, LP 515 for Fo‐mClover3; RFP filter set: BP 540/580, FT 585, LP 590 for Fo‐mRuby3). Fungal transformants displaying the brightest fluorescence intensity were selected for subsequent experimental analyses.

### 4.4 Conidial Germination Assays

Conidial germination assays were performed as described by Vitale et al. (2019). Briefly, fresh microconidia were harvested from 3‐ to 5‐day‐old PDB cultures, washed twice with sterile water, and adjusted to desired concentrations using haemocytometer counts. Germination was assessed at low concentration of inoculum (LCI; 3.2 × 10^6^ conidia/mL) and high concentration of inoculum (HCI; 8.6 × 10^7^ conidia/mL) in 1 mL of germination minimal medium (GMM; Vitale et al. 2019). Cultures were incubated at 28°C with 170 rpm shaking for 13 h (LCI) or 15 h (HCI). Samples were then vortexed to dissociate weakly adherent hyphae, transferred to microscope slides, and examined using a BH‐2 microscope (Olympus) with differential interference contrast at 400× magnification. At least 300 conidia per replicate were counted across three independent experiments, with germination defined as germ tube length exceeding conidial diameter. Results are expressed as percentage of germinated conidia.

### 4.5 VHF Assays

For quantification of VHF events, assays were performed following Nordzieke et al. (2019). Fresh microconidia (45 μL of 7 × 10^7^ conidia/mL suspension) were spread on plates containing 5 mL water agar (2% w/v) supplemented with 25 mM NaNO_3_ using sterile glass beads and incubated at 28°C for 14 h. Fusion events were quantified using a BH‐2 microscope (Olympus) with differential interference contrast at 400× magnification. Three hundred germlings per replicate were examined across three independent experiments, with fusion scored when continuous cytoplasmic connections were observed between adjacent germlings through CATs. Results are expressed as percentage of germlings participating in fusion events.

To visualise VHF events, including rare fusion occurrences occurring both intrastrain (within the same *MAT* isolate) and interstrain (between different *MAT* isolates), fluorescently labelled strains of MN25 and 4287 expressing Fo‐mClover3 or Fo‐mRuby3 were employed using a modified protocol. For fluorescence microscopy analysis, equal volumes (22.5 μL each) of differently labelled strains at 7 × 10^7^ conidia/mL were co‐inoculated to achieve the same total conidial density while enabling discrimination of fusion partners through distinct fluorescent signals. Fusion events between strains were assessed using appropriate filter sets on an Axio Imager M2 microscope (Zeiss), with successful fusion events identified by the presence of both fluorescent signals within continuous hyphal networks, confirming cytoplasmic continuity between genetically distinct isolates.

Rare fusion events in the MN25 isolate were further confirmed by staining with 10 μg/mL of the membrane‐impermeant dye propidium iodide (PI) applied 30 min prior to microscopic examination to assess cell wall continuity between adjacent fusing germlings, as previously described by Turrà et al. (2016). Imaging was performed using appropriate filter sets (GFP filter set: BP 450/490, FT 510, LP 515 for Fo‐mClover3; RFP filter set: BP 540/580, FT 585, LP 590 for Fo‐mRuby3; PI filter set: BP 535/550, FT 570, LP 590 for propidium iodide) on an Axio Imager M2 microscope (Zeiss) with differential interference contrast capabilities.

### 4.6 Hyphal Network and Aggregate Formation Analysis

Hyphal aggregate formation was evaluated following the methodology of Segorbe et al. (2017) with minor modifications. Fresh microconidia (100 μL of 1 × 10^8^ conidia/mL) were inoculated in glass tubes with 2 mL of minimal medium (MM; Puhalla 1968) supplemented with 3% sucrose and 50 mM NaNO_3_ and incubated at 28°C with 170 rpm orbital shaking for 48 h. Following incubation, samples were transferred to 24‐well plates for microscopic examination. Aggregate morphology and structural characteristics were documented using a binocular microscope (Leica Microsistemas S.L.U.) equipped with a Leica DC 300F digital camera. Multicellular aggregates were defined as hyphal networks exhibiting visible three‐dimensional organisation, and morphological features were systematically recorded through high‐resolution digital photography.

### 4.7 RNA Extraction and Quantitative Gene Expression Analysis

For gene expression analysis, fresh microconidia (5 × 10^6^) were initially germinated in PDB for 14 h at 28°C. Germlings were subsequently transferred to 200 mL of GMM supplemented with 25 mM sodium glutamate and 20 mM 4‐(2‐hydroxyethyl)‐1‐piperazineethanesulfonic acid (HEPES), adjusted to pH 7.4, and incubated for an additional 14 h. To induce high‐density conditions that simulate APS scenarios, cultures were concentrated 5‐fold by resuspending harvested germlings in GMM containing 5 mM sodium glutamate and incubated for 1 h following the protocol of Vitale et al. (2019). Mycelium was harvested by vacuum filtration, immediately flash‐frozen in liquid nitrogen, and stored at −80°C until RNA extraction.

RNA extraction was performed using the TRIzol reagent (Invitrogen) according to the manufacturer's instructions. RNA quality and concentration were determined by NanoDrop ND‐1000 spectrophotometry and agarose gel electrophoresis.

Complementary DNA synthesis was carried out using Moloney Murine Leukaemia Virus reverse transcriptase with 1 μg of total RNA and poly‐d(T) antisense primers according to the supplier's protocol (Invitrogen). Quantitative PCR was performed using an iQ SYBR Green Supermix (Bio‐Rad) in an iCycler apparatus (Bio‐Rad) with 15 μL reactions containing 7.5 μL SYBR Green Supermix, 400 ng cDNA template, and 300 nM each gene‐specific primer as described previously (Vitale et al. 2019). Relative transcript levels were calculated using the comparative threshold cycle (ΔΔ*C*_t_) method with peptidyl‐prolyl isomerase (*ppi*) as the reference gene. All reactions were performed with three biological replicates and technical duplicates.

### 4.8 Plant Pathogenicity Assays

Tomato seeds (cv. Monika) were surface sterilised in 20% (v/v) sodium hypochlorite for 20 min, washed three times with sterile water for 10 min each, and planted in sterile vermiculite. Seedlings were grown in plant growth chambers at 28°C with 12‐h photoperiods and ~70% of humidity until 14 days old. Root‐dip inoculation was performed by immersing seedling roots in a microconidia suspension (5 × 10^6^ conidia/mL in sterile water) for 30 min, followed by transplanting into sterile vermiculite and maintenance under identical growth chamber conditions. Control plants were mock‐inoculated with sterile water using the same protocol. Disease progression and plant mortality were monitored and recorded daily over a 30‐day observation period. Survival data were analysed using the Kaplan–Meier method, with statistical comparisons between treatment groups performed using the log‐rank (Mantel–Cox) test. All statistical analyses were conducted using GraphPad Prism 6.0 software. Each treatment group comprised 15 individual plants, and the complete experimental protocol was replicated on three independent occasions to ensure reproducibility.

### 4.9 Statistical Analysis

Data from spore germination and vegetative hyphal fusion assays was analysed using Yates' corrected chi‐squared test (two‐sided). Plant survival data were analysed using Kaplan–Meier survival analysis with log‐rank (Mantel–Cox) test for comparing survival curves between treatment groups. Gene expression data were assessed using Welch's *t*‐test (two‐tailed). Statistical analyses were performed using GraphPad Prism 6.0 software with significance threshold set at *p* < 0.05. All experiments included appropriate controls and were performed with biological replicates as specified for each assay type.

### 4.10 Bioinformatic Analysis of *MAT* Locus Structure and Protein Domain Architecture

Complete *MAT1‐1* and *MAT1‐2* locus sequences, including flanking regions, were obtained from the NCBI Genome Database (GenBank accessions AGBH00000000 and AAXH00000000) and the MycoCosm database. Protein domain prediction was carried out using sequence similarity searches against the UniProt database and motif analysis via the PROSITE database (ExPASy; https://prosite.expasy.org) and through the InterProScan (https://www.ebi.ac.uk/interpro/) and NCBI Conserved Domain Search tools (https://www.ncbi.nlm.nih.gov/Structure/cdd/wrpsb.cgi), to identify conserved domains associated with mating‐type proteins. Multiple sequence alignments of protein and DNA sequences were carried out using Clustal Omega via the EMBL‐EBI web server (https://www.ebi.ac.uk/Tools/msa/clustalo/).

## Author Contributions

**Stefania Vitale:** conceptualisation, data curation, formal analysis, methodology, investigation, supervision, project administration, writing – review and editing, writing – original draft, validation. **Antonia Barberio:** methodology, formal analysis. **Riccardo Cantelli:** methodology, formal analysis. **Marta Ranesi:** methodology, formal analysis. **Filippo De Curtis:** supervision, resources, writing – review and editing, project administration. **Antonio Di Pietro:** supervision, resources, writing – review and editing, project administration. **David Turrà:** conceptualisation, methodology, formal analysis, resources, data curation, writing – original draft, supervision, project administration, funding acquisition.

## Funding

This work was supported by grants from the Italian Ministry of University and Research (PRIN 2022‐TAKLPAT/2022SC83XK) to D.T. A.B. was supported by the Ph.D. program in Agri‐Food Production Sciences—University of Molise—Italy, under the supervision of F.D.C. This study was carried out within the Agritech National Research Center and received funding from the European Union Next‐Generation EU (PIANO NAZIONALE DI RIPRESA E RESILIENZA (PNRR)—MISSIONE 4 COMPONENTE 2, INVESTIMENTO 1.4–D.D. 1032 17/06/2022, CN00000022). This manuscript reflects only the authors' views and opinions, neither the European Union nor the European Commission can be considered responsible for them.

## Conflicts of Interest

The authors declare no conflicts of interest.

## Supporting information


**Figure S1:** Targeted deletion of the *Fusarium oxysporum* f. sp. *lycopersici MAT1‐1* locus.

**Figure S2:** Generation of *MAT1‐1* complemented strains in the *Fusarium oxysporum* f. sp. *lycopersici MAT1‐1*Δ genetic background.

**Figure S3:** Generation of *MAT1‐2* insertional mutants in the *Fusarium oxysporum* f. sp. *lycopersici MAT1‐1*Δ genetic background.

**Figure S4:** Generation of *MAT1‐1* + *MAT1‐2* insertional mutants in the *Fusarium oxysporum* f. sp. *lycopersici* 4287 genetic background.

**Figure S5:** Generation of *MAT1‐2* + *MAT1‐1* insertional mutants in the *Fusarium oxysporum* f. sp. *lycopersici* MN25 genetic background.

**Figure S6:** Strain‐specific expression patterns of pheromone signalling components in *Fusarium oxysporum* f. sp. *lycopersici* wild‐type isolates.

**Figure S7:** mpp70248‐sup‐0007‐FigureS7.tif. *MAT1‐2* insertion into wild‐type *Fusarium oxysporum* f. sp. *lycopersici* 4287 background reveals complex compensatory regulation of autocrine pheromone signalling networks.

**Figure S8:** Biphasic dose–response effects of *MAT1‐1* insertion in the *Fusarium oxysporum* f. sp. *lycopersici* MN25 genetic background.

## Data Availability

The data that support the findings of this study are available from the corresponding author upon reasonable request.
