# Supplementary figures and images for: Mating‐Type Loci Modulate Pathogenicity and Non‐Sexual Development Through Autocrine Pheromone Signalling in the Asexual Fungus Fusarium oxysporum
Source: Mol Plant Pathol. 2026 Mar 23;27(3):e70248. doi: 10.1111/mpp.70248 (PMC13097639; doi:10.1111/mpp.70248)

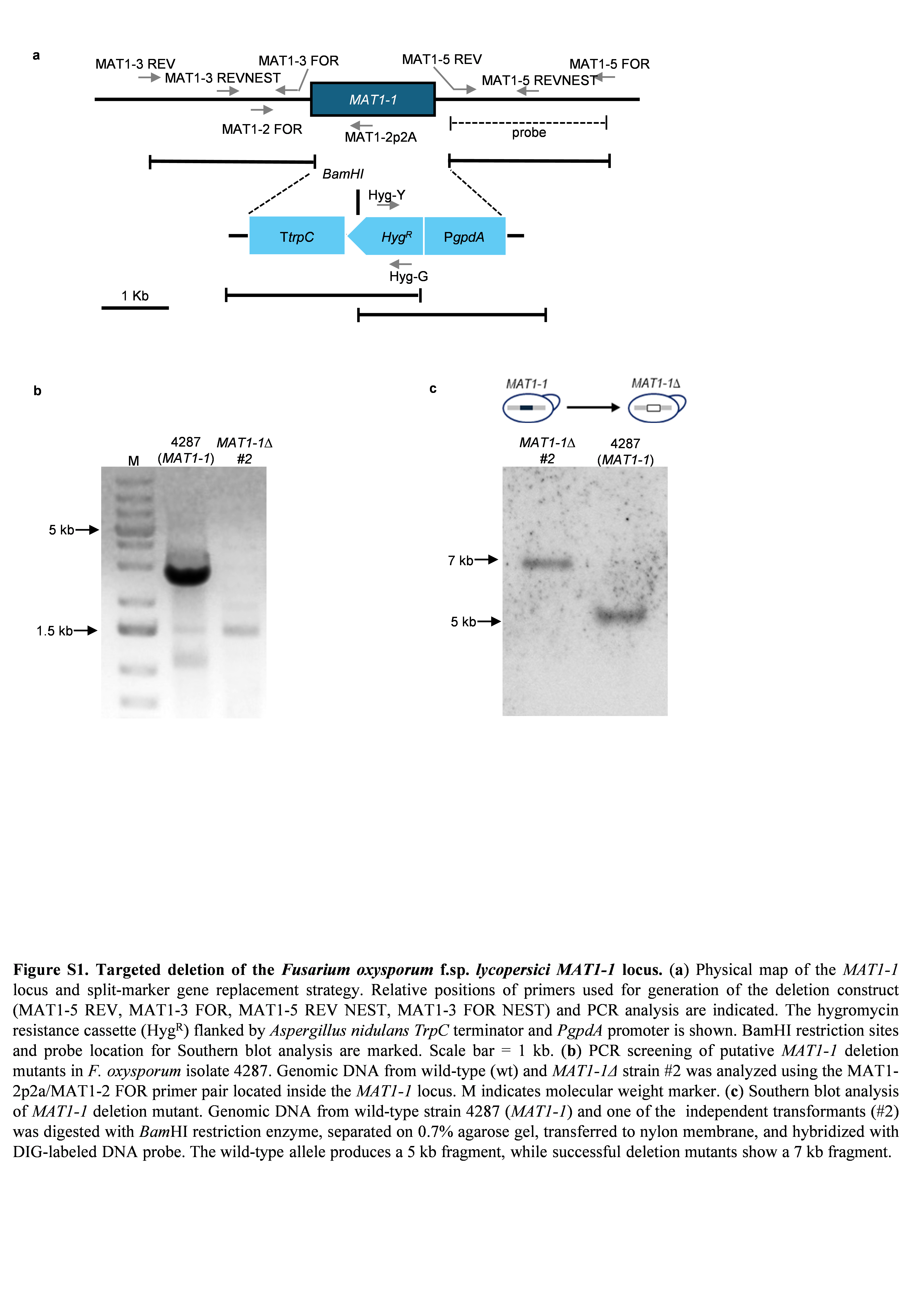

Supplement: Supplementary file 1 — Figure S1: Targeted deletion of the Fusarium oxysporum f. sp. lycopersici MAT1‐1 locus. [file MPP-27-e70248-s004.tif]

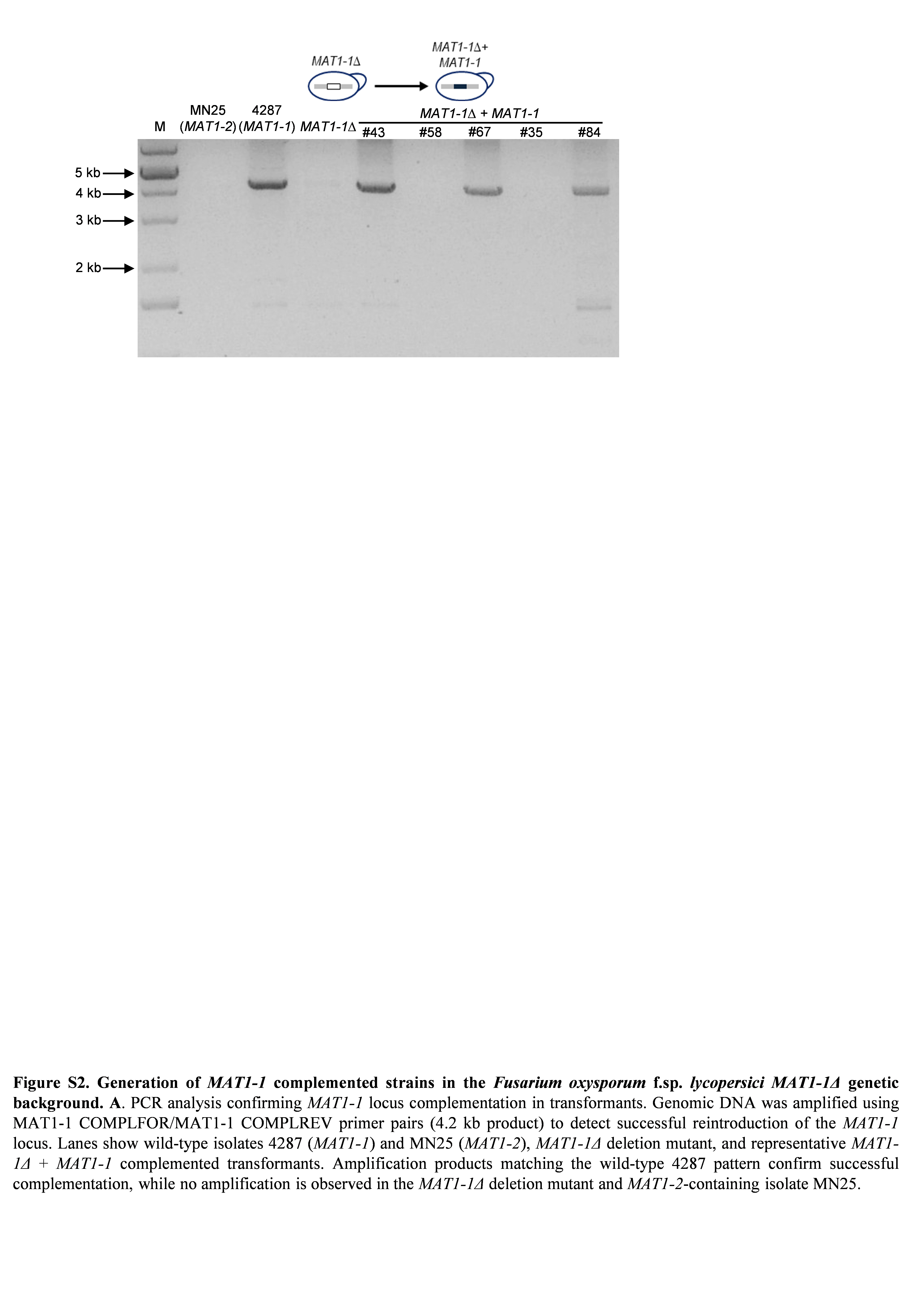

Supplement: Supplementary file 2 — Figure S2: Generation of MAT1‐1 complemented strains in the Fusarium oxysporum f. sp. lycopersici MAT1‐1Δ genetic background. [file MPP-27-e70248-s002.tif]

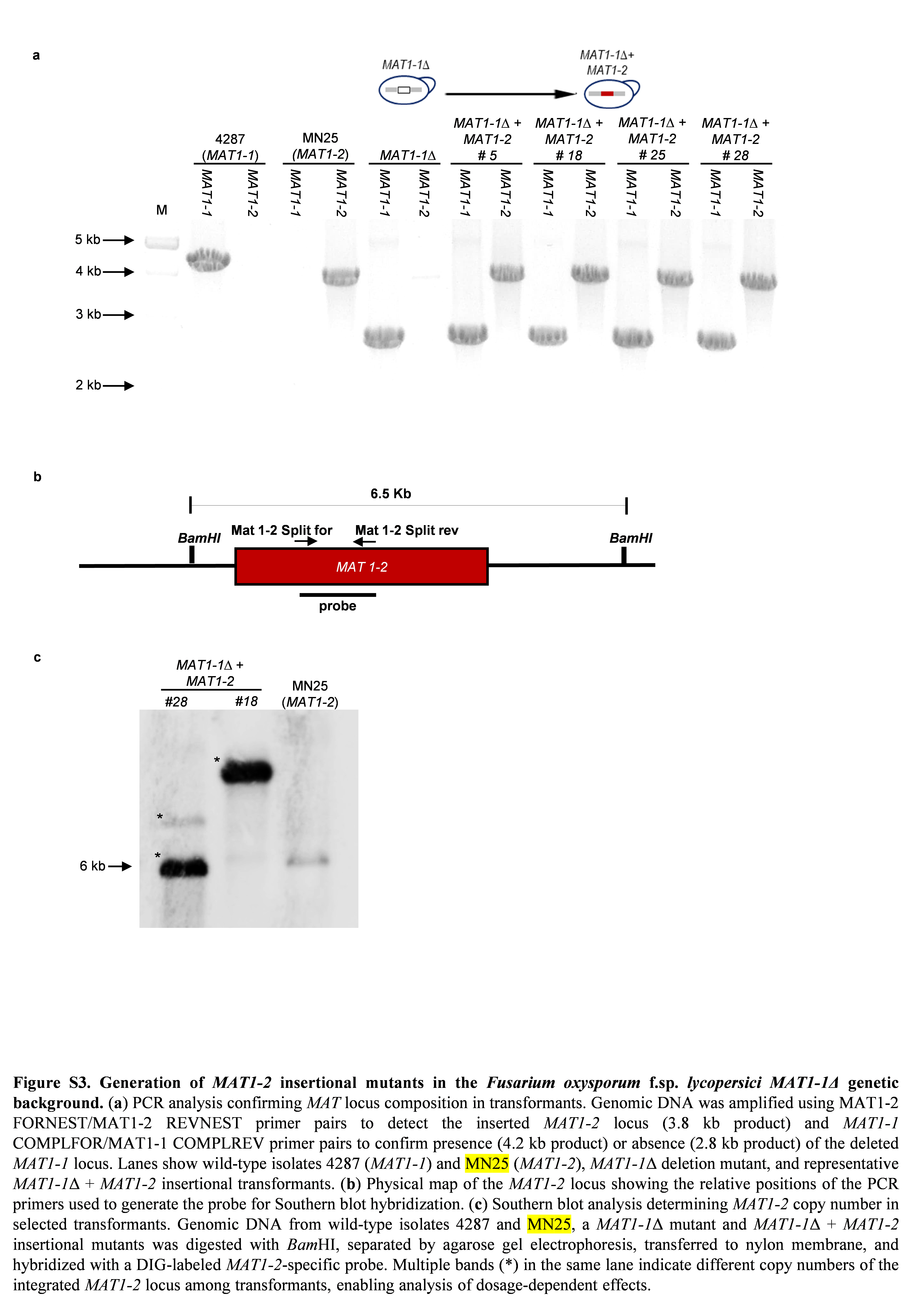

Supplement: Supplementary file 3 — Figure S3: Generation of MAT1‐2 insertional mutants in the Fusarium oxysporum f. sp. lycopersici MAT1‐1Δ genetic background. [file MPP-27-e70248-s005.tif]

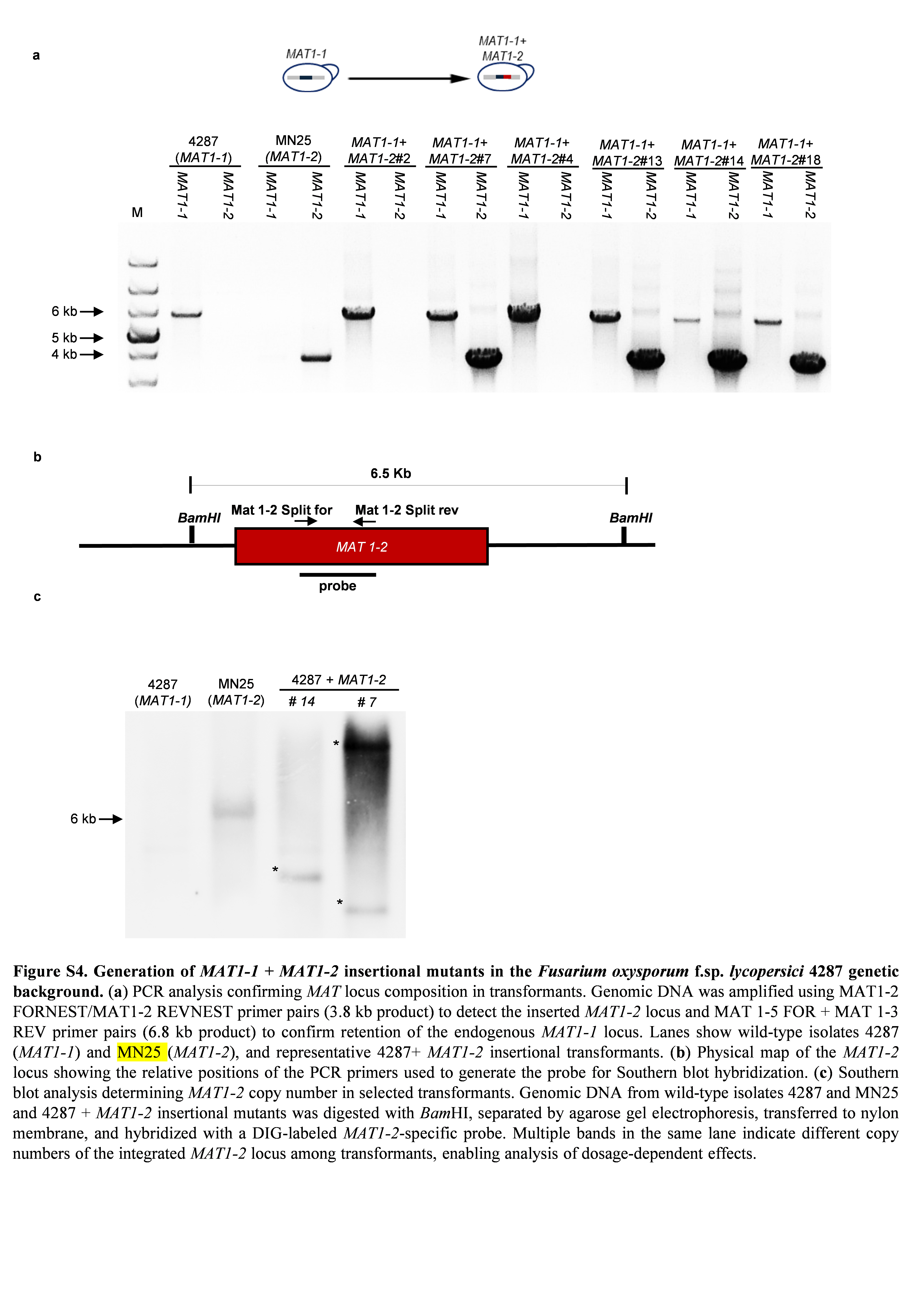

Supplement: Supplementary file 4 — Figure S4: Generation of MAT1‐1 + MAT1‐2 insertional mutants in the Fusarium oxysporum f. sp. lycopersici 4287 genetic background. [file MPP-27-e70248-s001.tif]

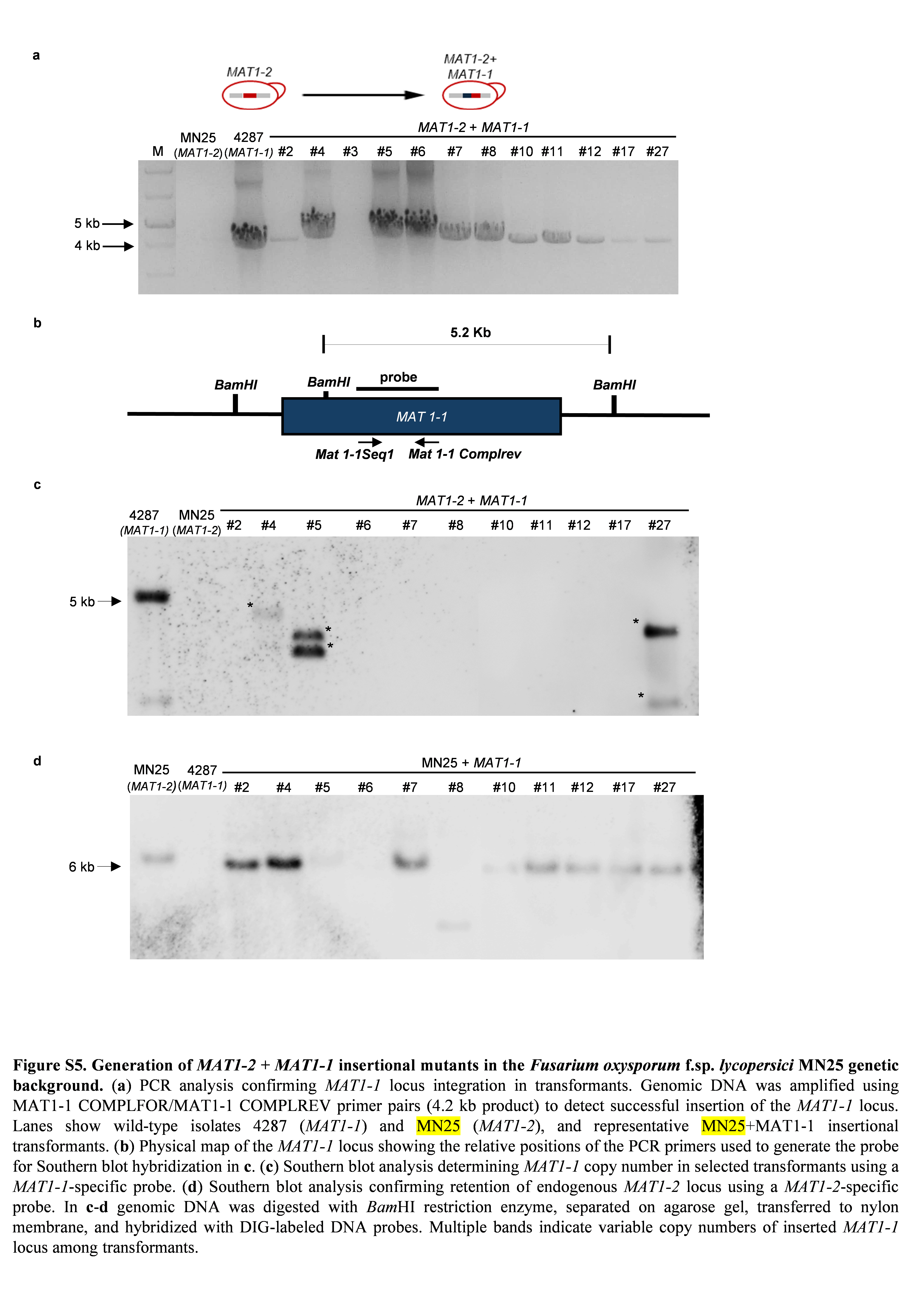

Supplement: Supplementary file 5 — Figure S5: Generation of MAT1‐2 + MAT1‐1 insertional mutants in the Fusarium oxysporum f. sp. lycopersici MN25 genetic background. [file MPP-27-e70248-s007.tif]

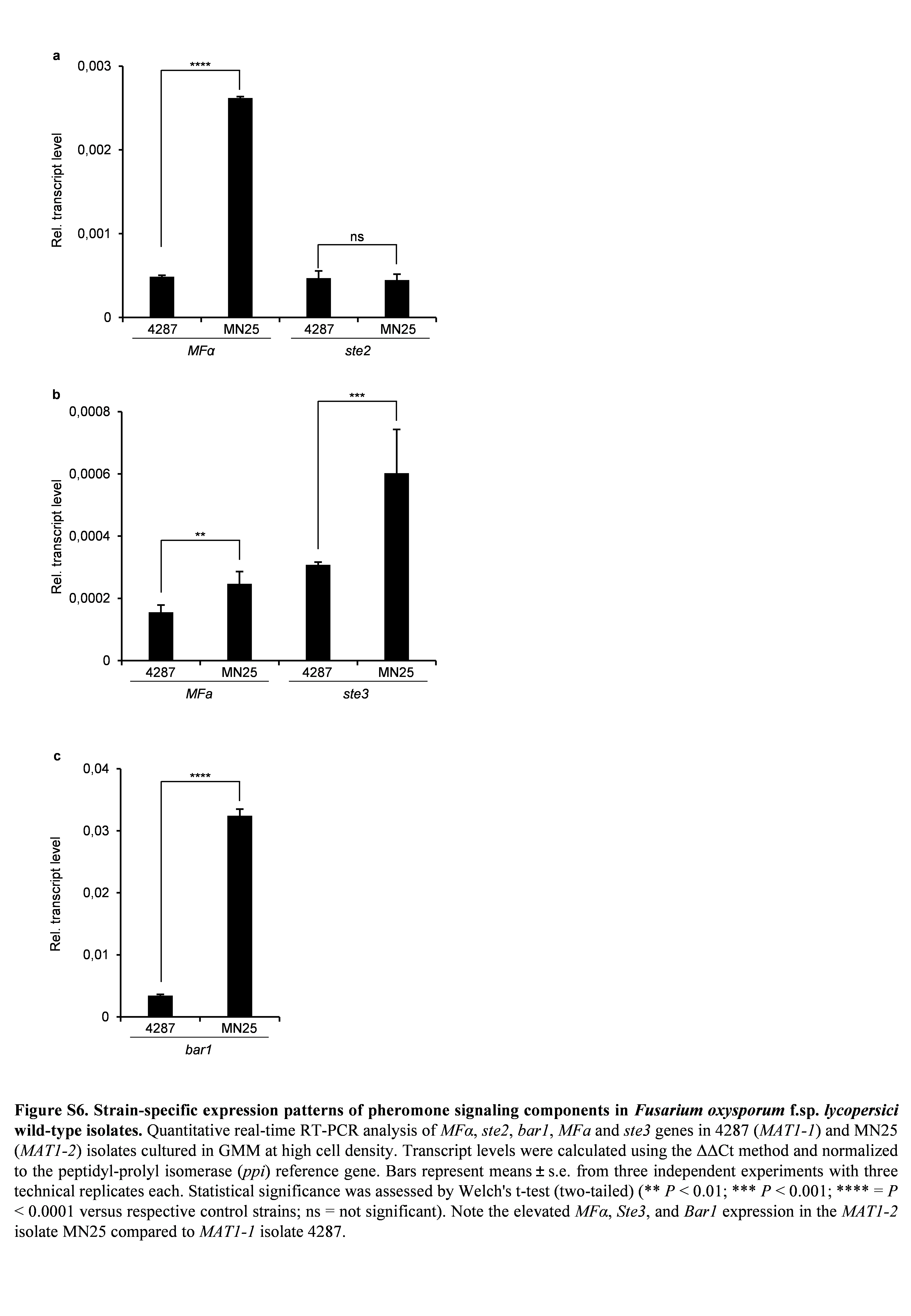

Supplement: Supplementary file 6 — Figure S6: Strain‐specific expression patterns of pheromone signalling components in Fusarium oxysporum f. sp. lycopersici wild‐type isolates. [file MPP-27-e70248-s003.tif]

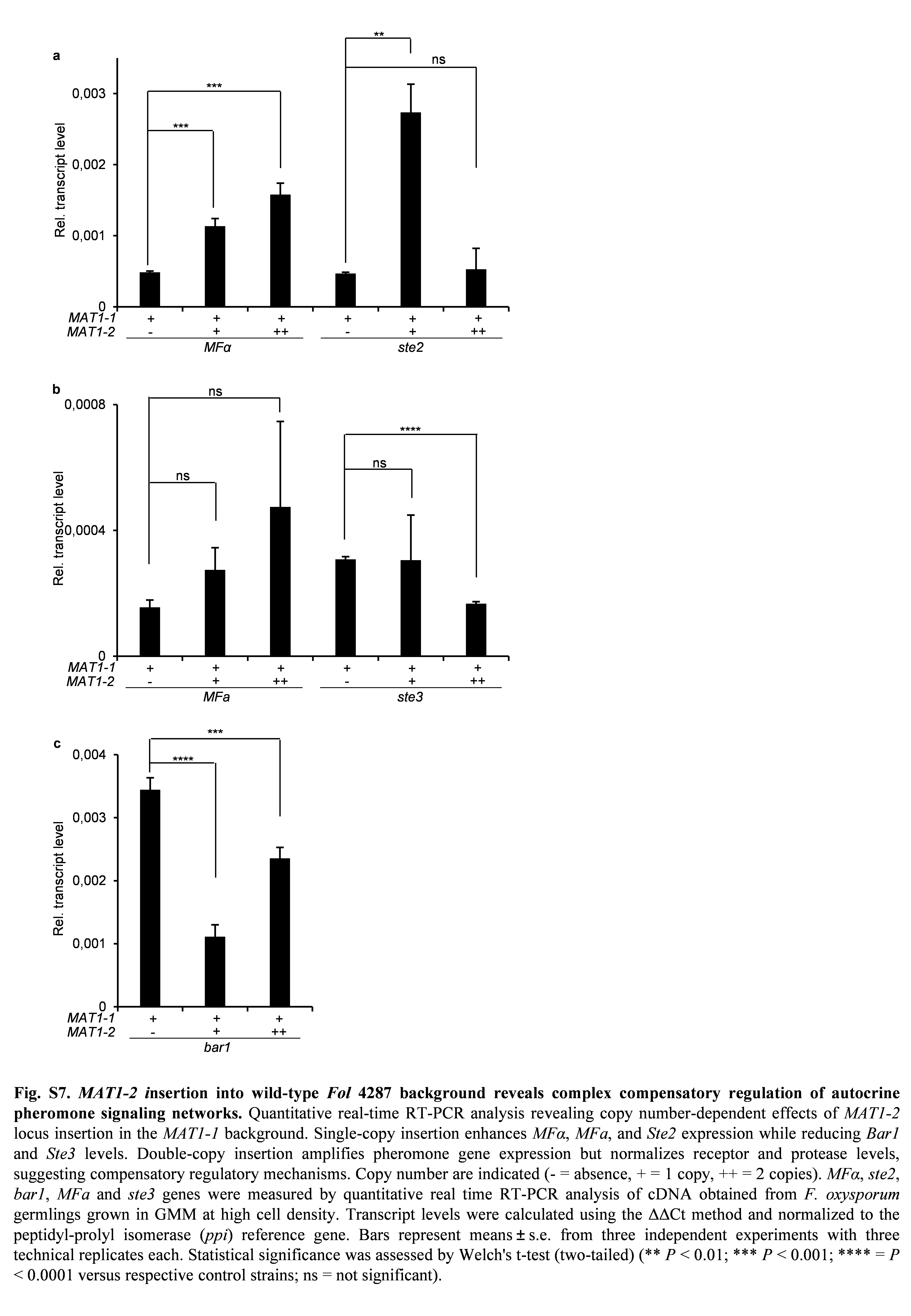

Supplement: Supplementary file 7 — Figure S7: mpp70248‐sup‐0007‐FigureS7.tif. MAT1‐2 insertion into wild‐type Fusarium oxysporum f. sp. lycopersici 4287 background reveals complex compensatory regulation of autocrine pheromone signalling networks. [file MPP-27-e70248-s006.tif]

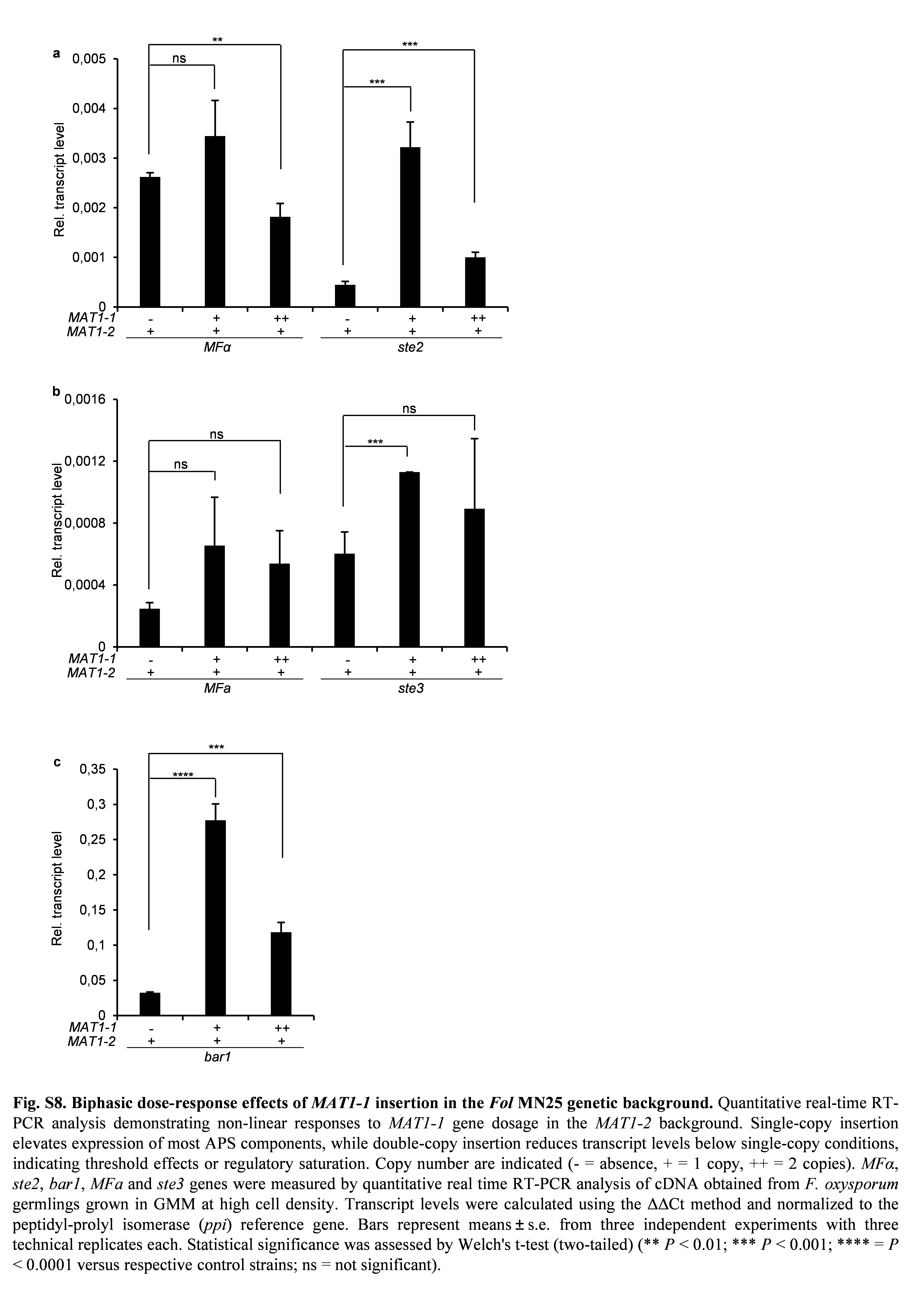

Supplement: Supplementary file 8 — Figure S8: Biphasic dose–response effects of MAT1‐1 insertion in the Fusarium oxysporum f. sp. lycopersici MN25 genetic background. [file MPP-27-e70248-s008.tif]
